# Supplementary material for: mRNA Cap Methyltransferase, RNMT-RAM, Promotes RNA Pol II-Dependent Transcription
Source: Cell Rep. 2018 May 2;23(5):1530–42. doi: 10.1016/j.celrep.2018.04.004 (PMC5946721; doi:10.1016/j.celrep.2018.04.004)
Supplement: Document S1. Figures S1–S9 and Table S1 [file mmc1.pdf]

Cell Reports, Volume *23*

Supplemental Information

mRNA Cap Methyltransferase, RNMT-RAM,

Promotes RNA Pol II-Dependent Transcription

Dhaval Varshney, Olivia Lombardi, Gabriele Schweikert, Sianadh Dunn, Olga Suska, and Victoria H. Cowling

Figure S1

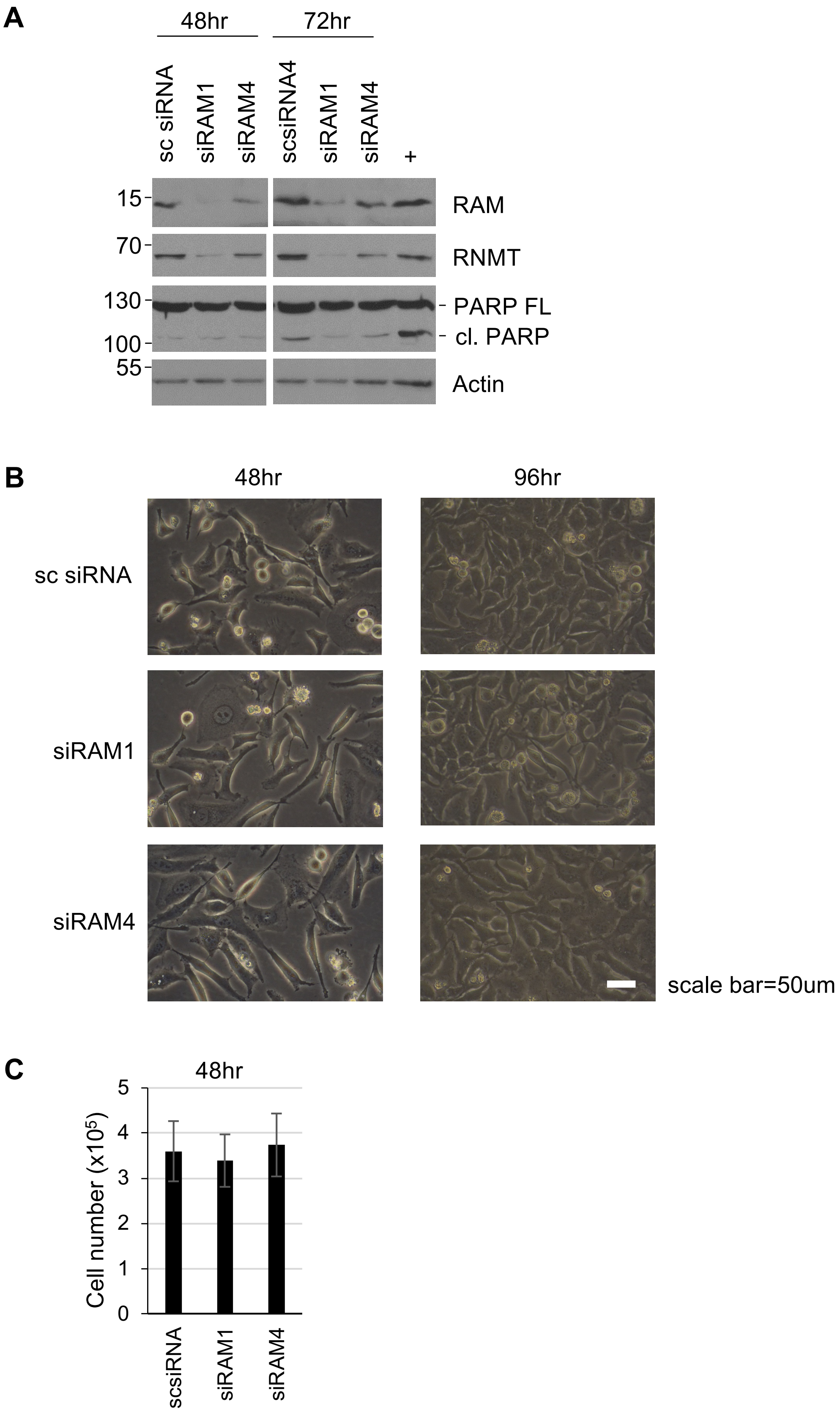

Figure S1, related to Figure 1: Impact of RAM depletion on cell number and apoptosis
HeLa cells were transfected with siRAM1, siRAM4 and sc siRNA (scrambled control). After time indicated, a) RAM expression and cleavage of PARP assessed by western blot. "+" indicates cells treated with 250um THZ-1 for 2 hours to induce apoptosis (representative of 2 independent experiments), b) phase-contrast images taken at 400X magnification (representative of 2 independent experiments), c) cells were counted (average and standard error presented for 3 independent experiments).

Figure S2

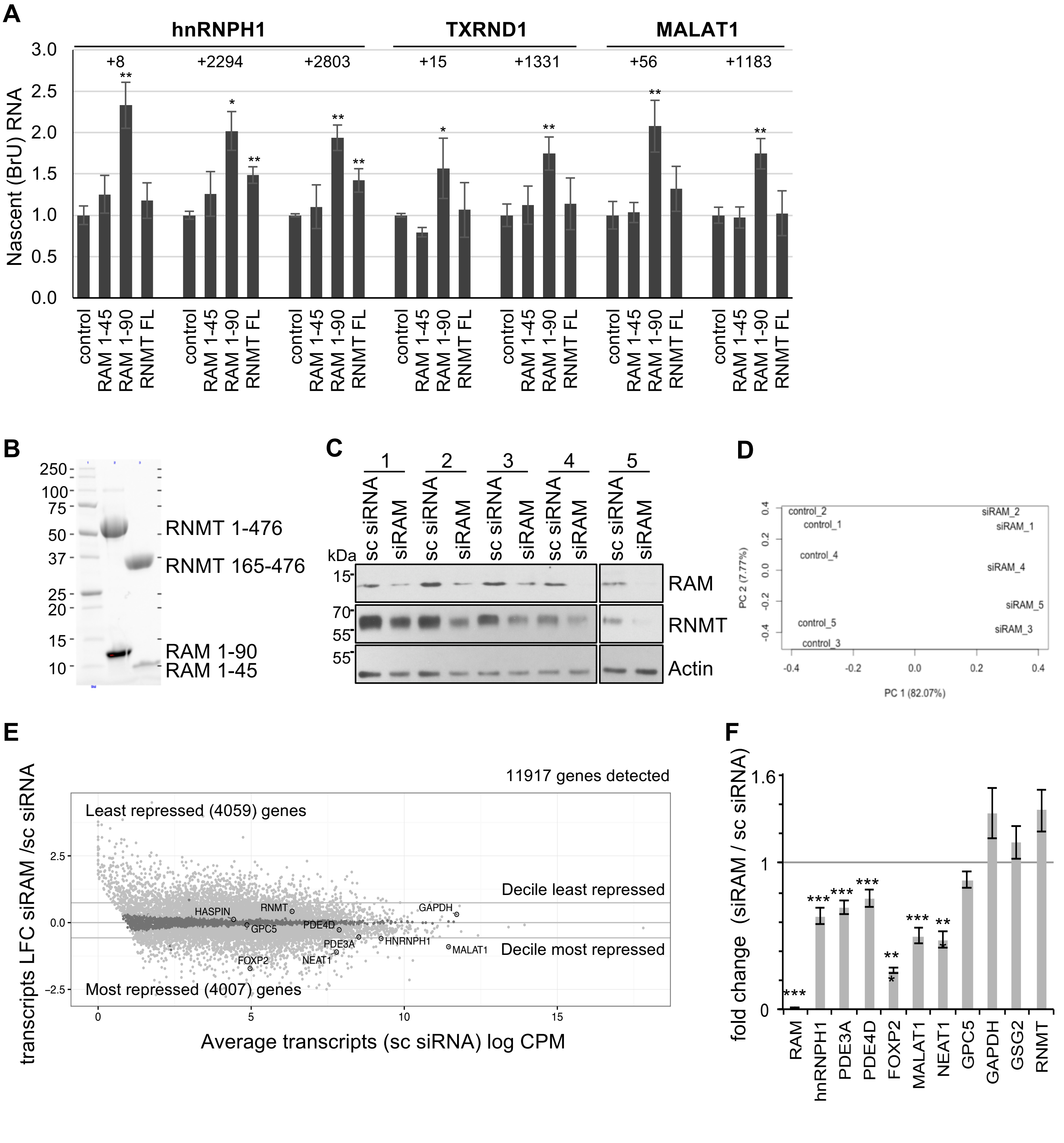

Figure S2, related to Figure 1. Impact of RAM on transcription.

a) HeLa nuclei were incubated with NTPs, BrUTP and recombinant RNMT, RAM 1-90 or RAM 1-45 for 20mins. Br-RNA purified and used as a substrate for PCR with primers indicated. Average and standard deviation presented. n=4. *, P < 0.05; **; P <0.01; Students t-test. b) SDS-PAGE analysis of recombinant RNMT-RAM proteins used in this study. c) HeLa cells transfected with siRAM 1 or sc siRNA for 36 hours, in five independent biological replicates. Western blot analysis. d) Principal components analysis of RNA sequencing performed. e) RNA sequencing analysis. Scatter plot of differential transcript expression following RAM siRNA or sc siRNA transfection for 36hrs (n=5). For each annotated gene locus, $\log_2$ fold change (LFC siRAM/sc siRNA) expression plotted against expression in control cells ($\log_2$ counts per million (logCPM) of uniquely aligned reads). Deciles most and least repressed indicated. Most repressed genes () and least repressed genes shaded lighter and annotated. f) HeLa cells transfected with siRAM1 or sc siRNA for 36hrs. RTPCR analysis of mRNA indicated following . n=3 ***, P <0.005; Students t-test.

Figure S3

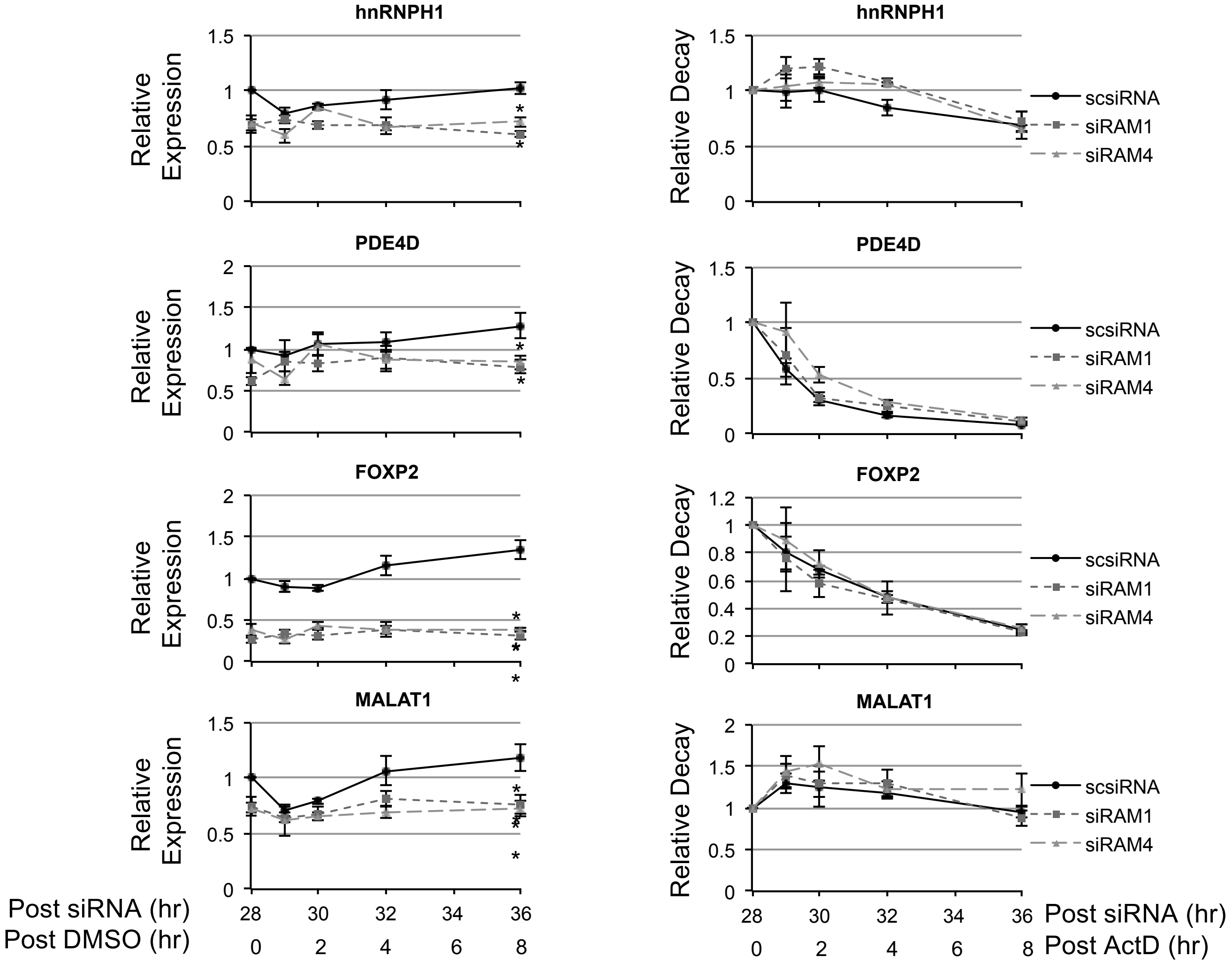

Figure S3, related to Figure 1. Gene expression analysis following RAM siRNA transfection Hela cells transfected with RAM siRNA or non-targeting control. 28 hours post-transfection 3μg/μl Actinomycin D or DMSO control added for further 8 hours. Mature transcript level determined by RTPCR. DMSO control data (left panels) normalised to 0 hour scrambled control. Actinomycin D data normalised to 0 hour samples for each siRNA (right panels).

Figure S4

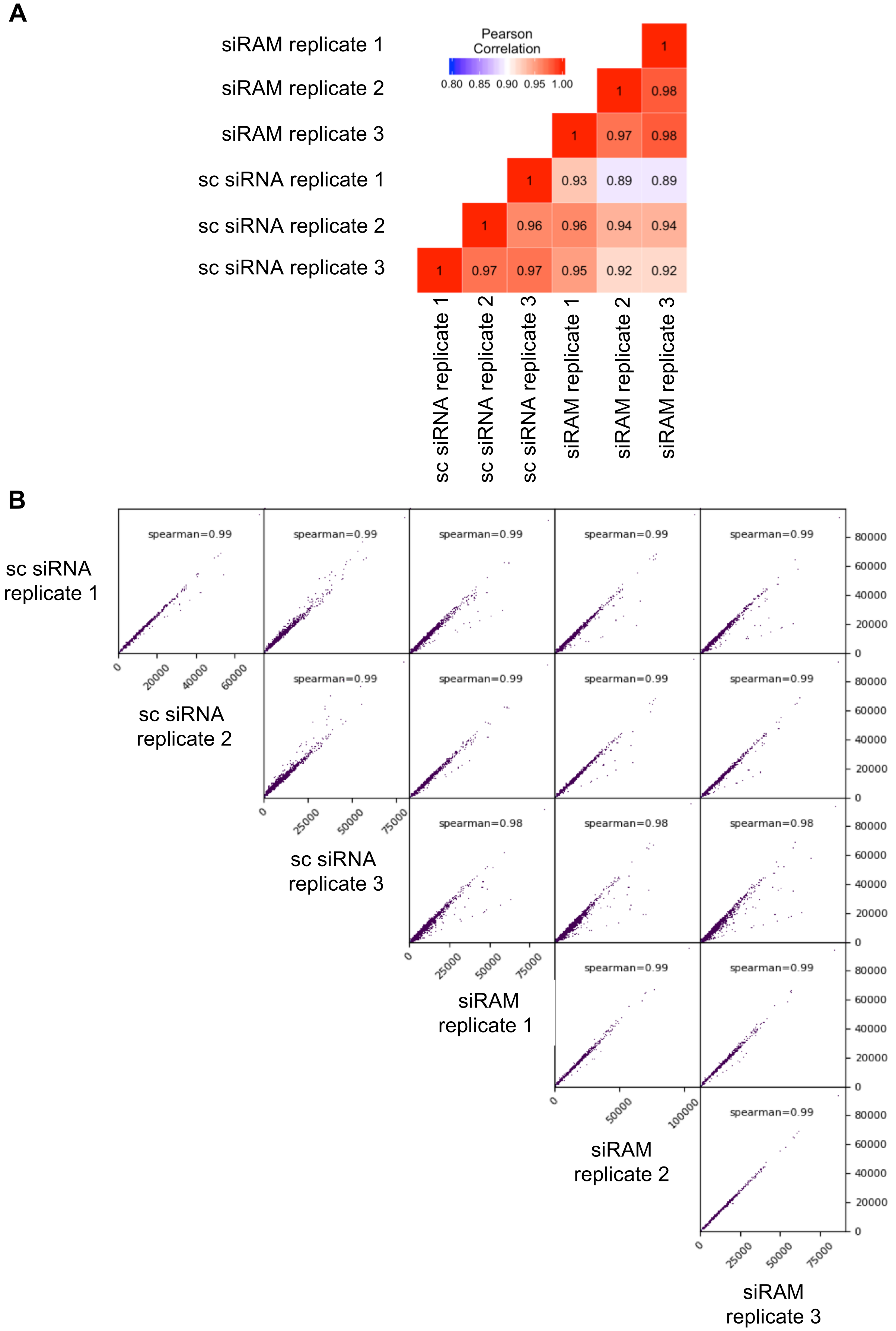

Figure S4, related to Figure 2. Correlation of RNA Pol II ChIP-Seq replicates

A) Heatmap for Pearson correlation of individual RNA pol II ChIP replicates from HeLa cells transfected with siRAM 1 or sc siRNA for 36 hours. Genome wide correlation for tag alignment to 10Kb bins. B) Scatterplots for RNA pol II ChIP replicates showing the total number of tags uniquely aligning to individual annotated loci. Calculated Spearman correlation values shown.

Figure S5

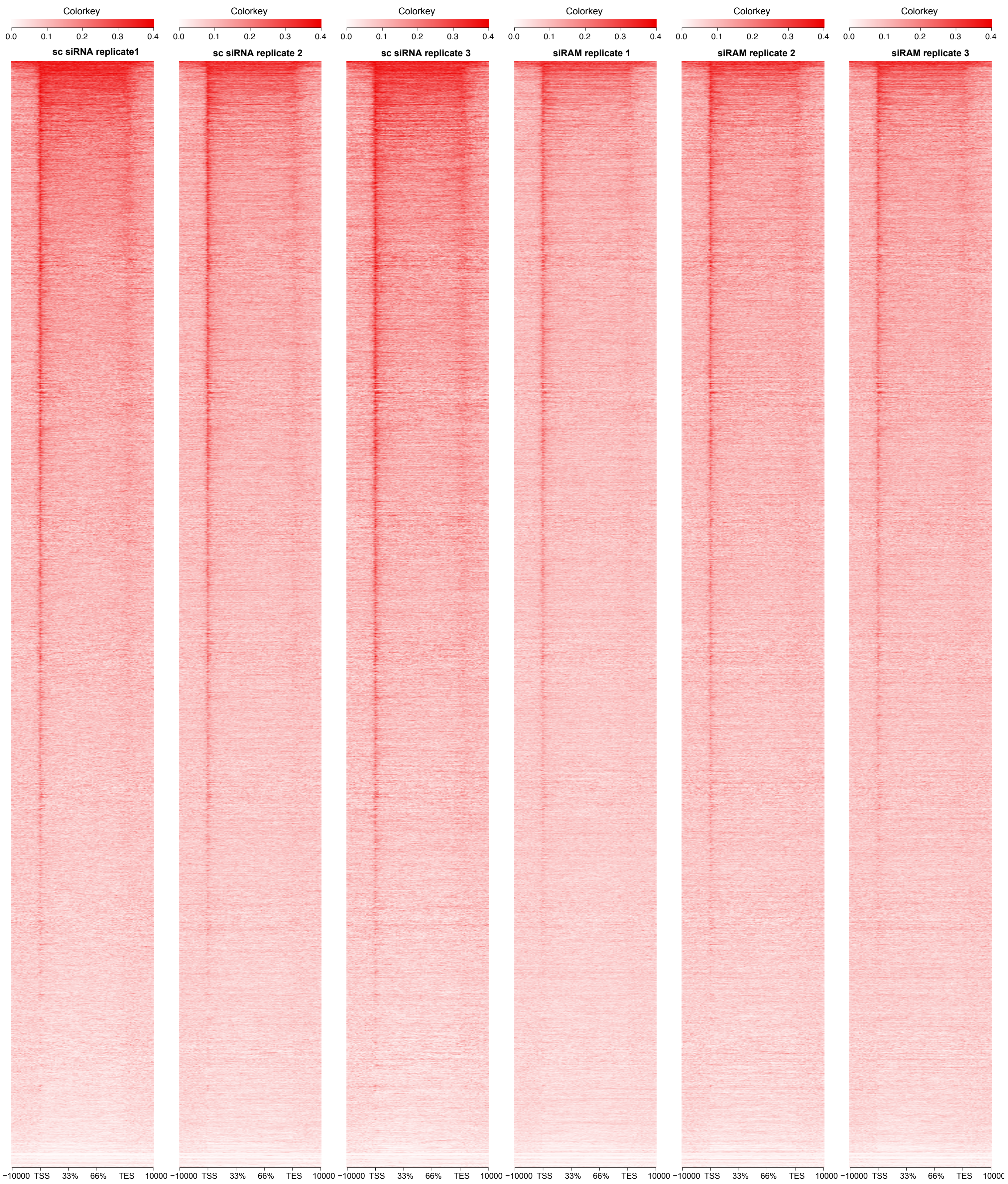

Figure S5, related to Figure 2. Heatmaps for RNA pol II ChIP replicates over gene bodies
Gene body heatmaps for all annotated gene loci in human genome assembly hg38 produced using ngs.plot (Shen et al., 2014)

Figure S6

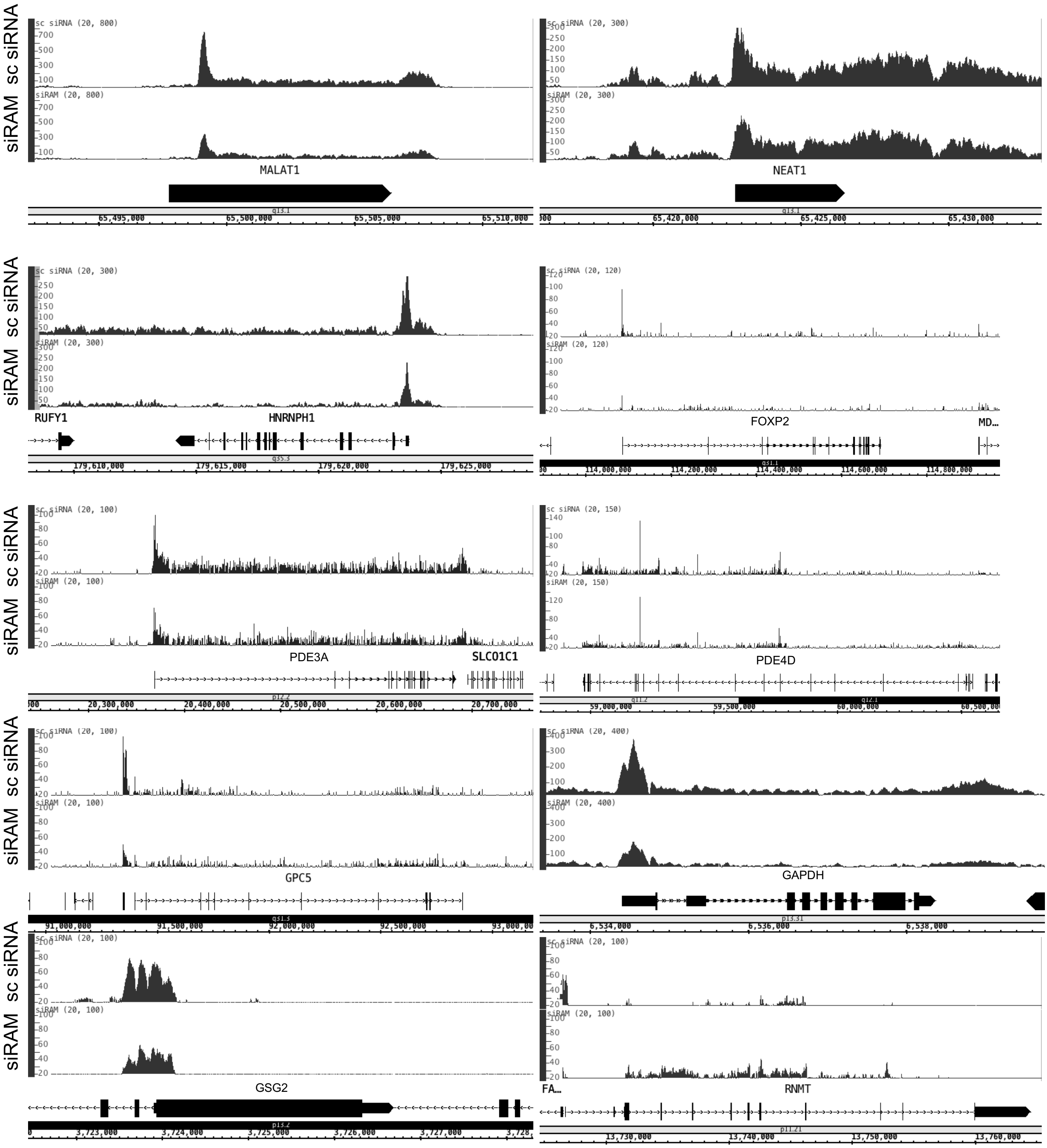

Figure S6, related to Figure 2. RNA Pol II ChIP signal at individual gene loci
Snapshot of RNA pol II ChIP tag distribution over individual gene loci following sc siRNA or si RAM transfection. Profiles created using pooled data from three replicates. Minimum cutoff of 20 tags.

A

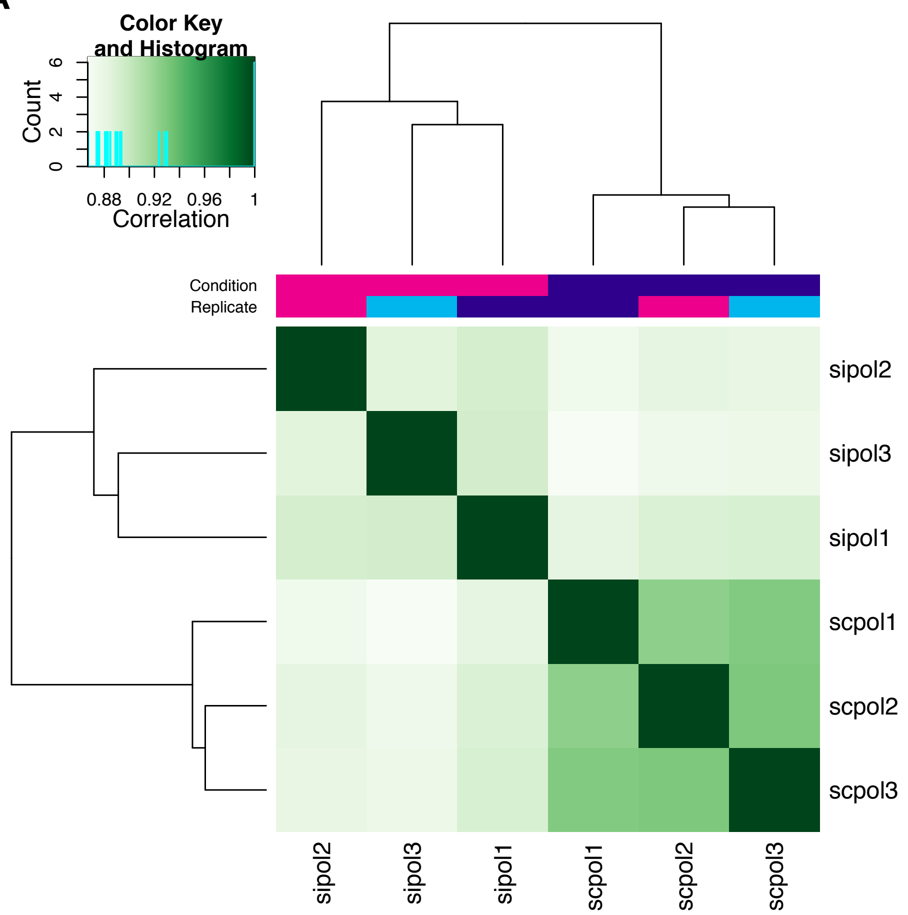

B

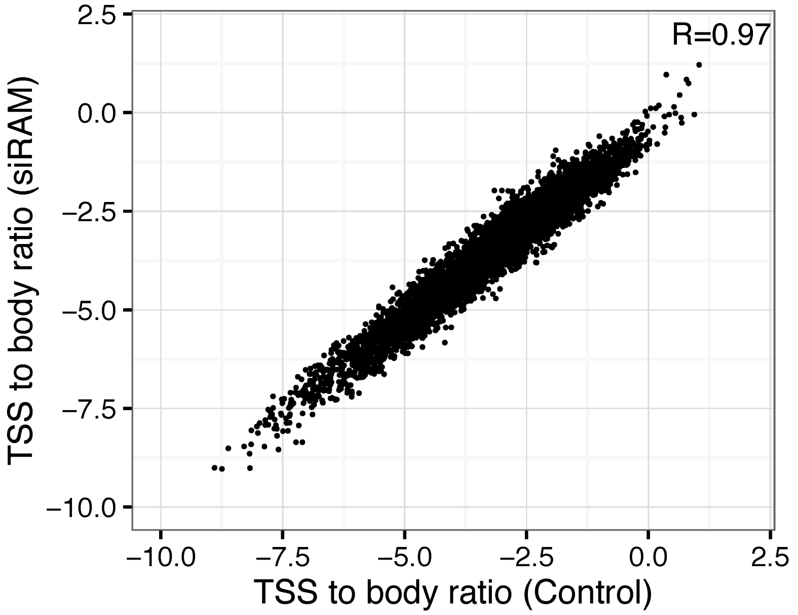

Figure S7, related to Figure 2. RAM depletion results in RNA pol II depletion

a) Correlation heatmap for peaks called from three independent biological replicates for RNA pol II occupancy in sc siRNA (sc) and siRAM (si) transfected cells. b) Correlation of Log_2 CPM TSS/gene body ratios of RNA pol II occupancy in sc siRNA (control) and siRAM transfected cells. R value represents Pearson's correlation.

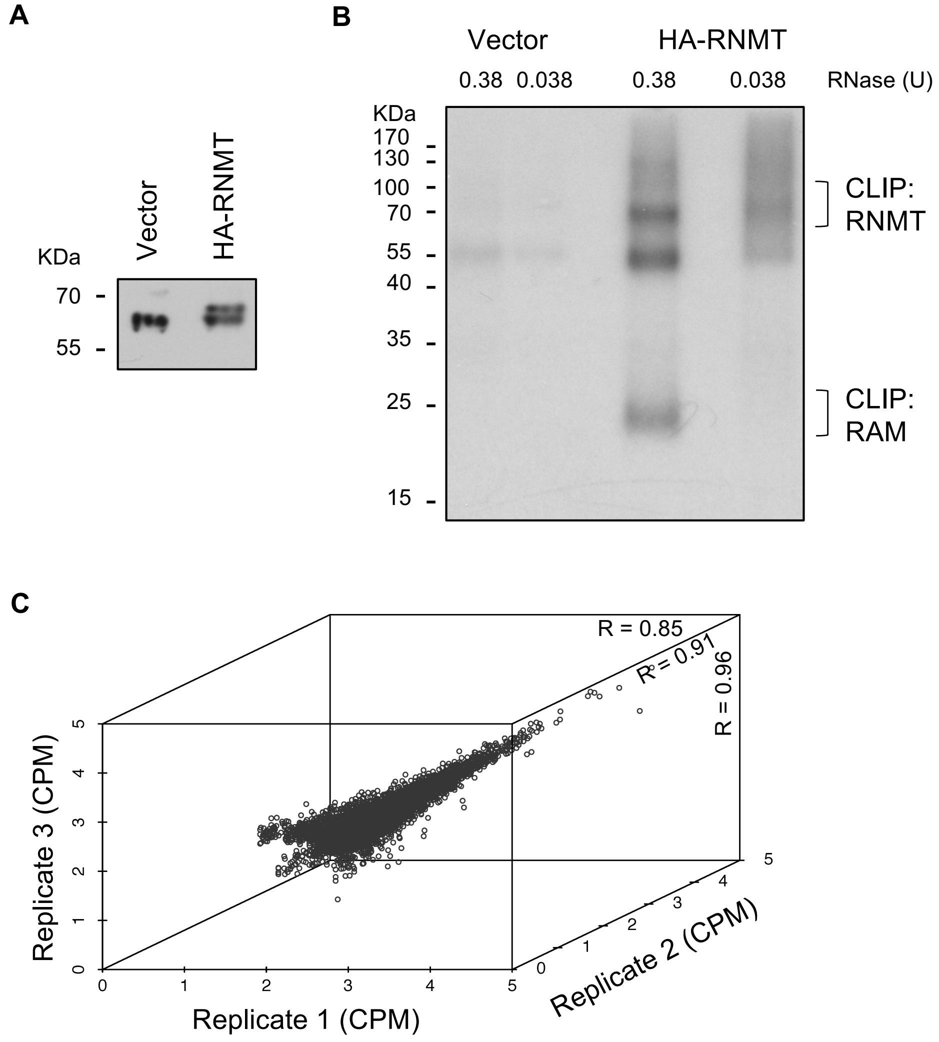

Figure S8, related to Figure 3. HA-RNMT expression and RNMT-CLIP

a) Western blot analysis of RNMT expression in HA-RNMT-expressing and vector control HeLa cells. The lower band is endogenous RNMT, the upper band is HA-RNMT. b) CLIP assay performed on HeLa cells stably expressing HA-RNMT or vector control. Immunoprecipitation performed with anti-HA antibody after RNase treatment. Reduced RNase concentration resulted in smearing of the ^{32}P-RNMT band and reduced intensity of the ^{32}P-RAM band. RAM is likely to be tethered to RNMT via RNA, migrating in a large RNMT-RAM-RNA complex. RNA from smear between 70 and 100 kDa was isolated for sequencing. c) Following RNA sequencing analysis, scatter plot of total counts per million (CPM) uniquely aligned reads per annotated gene locus, for three HA-RNMT CLIP replicates. Correlation scores on xy, yz and xz planes.

Figure S9

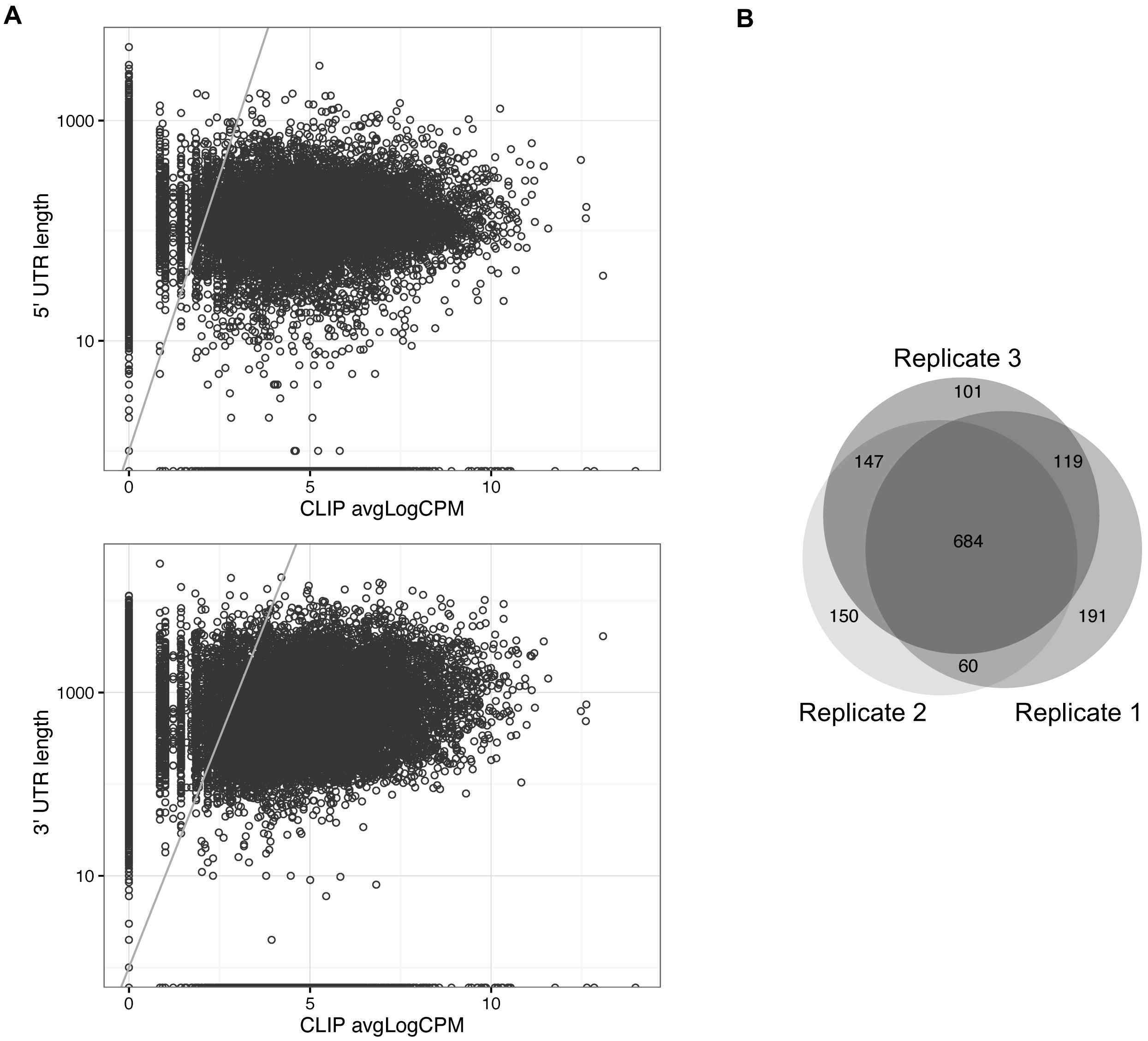

Figure S9, related to Figure 6. Correlation of RNMT CLIP signal and UTR length
a) Average Log_2 CPM from RNMT CLIP signal from three independent biological replicates plotted against the maximal annotated UTR length for each gene model. b) Venn diagram of top quartiles for three RNMT CLIP replicates. Intersection of annotated gene loci with maximum number of uniquely aligned reads denoted “RNMT-enriched targets”.

Table S1, related to Figures 1 and 5. Oligo sequences used for RT-qPCR analysis and CLIP library preparation

Target	Primer	Sequence
Actin	F	CCCAGAGCAAGAGAGG
Actin	R	GTCCAGACGCAGGATG
FOXP2	F	CACTTCCTGTCCAATCGCTG
FOXP2 mature	R	TTGGAGGAGGTAGTCGAGGA
FOXP2 nascent	R	GCCACCATGTTAACAATCACG
GAPDH	F	GGAGTCAACGGATTTGG
GAPDH	R	GTAGTTGAGGTCAATGAAGGG
GPC5	F	GGAGAGATATCAGATTGCGGC
GPC5	R	ACATAACACGGCCAGTCCTT
Haspin	F	GTCCGACGATCCTGACGAT
Haspin	R	TTTGGGGTCACGGTCAGG
hnRNPH1	F	GGTGTTGAAGCATACTGGTCC
hnRNPH1 mature	R	GTCCACCGGCAATGTTATCC
hnRNPH1 nascent	R	GCTAGCCAAAACTCACTGCT
MALAT1	F	GACGGAGGTTGAGATGAAGC
MALAT1	R	ATTCGGGGCTCTGTAGTCCT
NEAT1	F	TTGTTCCAGAGCCCATGAA
NEAT1	R	TGAAAACCTTTACCCCAGGA
PDE3A	F	CAGCAGCAAGTCCCATCG
PDE3A mature	R	CTGTGATCCTCTTGGCCCTC
PDE3A nascent	R	CTTCCCTTTAGTTCCCAGCG
PDE4D	F	CATGTGCAACCAACCATCCA
PDE4D mature	R	TAGGGTCTCTAGCTGGTCCA
PDE4D nascent	R	TGACTCTTGGCAGAAATGGT
RAM	F	CCTCAAACCTTTGGGATT
RAM	R	TTCCTGATACTCCTTGTCA
RNMT	F	GTACTGATATTGCCGATGT
RNMT	R	ATTCACTATCACGACGATT
CLIP - RT Primer		CNNNNGATCGTCGGACTGTAGAACTCTG AACGGATCCCTTGGCACC
Cut Oligo		CCAAGGGATCCGTTCAGAGTTCTAAAA
HNRNPH1 NRO 8-77	F	ACCTTATTTAGGTTGCGCAG
HNRNPH1 NRO 8-77	R	ACCTAGACACGCGACTTCTG
HNRNPH1 NRO 2294-2363	F	AACGGTTGTTTTCTTTCCAGACT
HNRNPH1 NRO 2294-2363	R	TCTGGTGTAGATGAAACGAATACCT
HNRNPH1 NRO 2803-2873	F	GTTCAGTTCTTCTCAGGTATGTAGT
HNRNPH1 NRO 2803-2873	R	CCTTGCCATAAGCTAGCCAAA
MALAT1 56-126	F	TTTTAGCAACGCAGAAGCCC
MALAT1 56-126	R	GAGGCGTCAGAGGGGAC
MALAT1 1183-1253	F	ACTACTTTTTGCCTCCCTCAC
MALAT1 1183-1253	R	ACGGAGAACAACTCGCATCA
TXNRD1 15-85	F	CTTGTGCGACATGGGCTG
TXNRD1 15-85	R	TTTCGTCTGCAGCTCCGTT
TXNRD1 1331-1401	F	CAAGGTCAGGCAAGGTCCA

TXNRD1 1331-1401	R	CCTGCTCAAACTCCTTCATTGT
HNRNPH1+1	F	GTGGGCTGGCTCTTGAAATT
HNRNPH1+1	R	GGCTAAGACGAAATGGCCAG
HNRNPH1+500	F	CTCTCAGTTCTGGTCCTCCC
HNRNPH1+500	R	GTGCCCAACATCATCGTCTC
HNRNPH1+1000	F	GCAGGTTTGGCAGACTTTGT
HNRNPH1+1000	R	AGCTTGGGTGACAGAGACTC
FOXP2+1	F	CTTTGTCACCCCTCACGTTG
FOXP2+1	R	GTGATTGTTTTGGCGTCCGT
FOXP2+500	F	AGAGAGGGGTGGGATTTTACG
FOXP2+500	R	GCTTTTCCCTCGCAAACAGA
FOXP2+1000	F	AGAACCTGGGATCCTATTGGG
FOXP2+1000	R	CCCTCATAGCTTGTTCATGCA
PDE3A+1	F	TGAGCGTGCTAGCCTTTAAC
PDE3A+1	R	GCCCACAATCCACTGAAAGT
PDE3A+500	F	TTCTGGATGGGCTTGTACCT
PDE3A+500	R	CGCTAGTCAAGGCGATCAT
PDE3A+1000	F	GAGGGAACAGGTAAGCACTG
PDE3A+1000	R	CTTCCCTTTAGTTCCCAGCG
GAPDH+1	F	TACTAGCGGTTTTACGGGC
GAPDH+1	R	GCTGCGGGCTCAATTTATAG
GAPDH+500	F	GAACCGCGTCTACGAGC
GAPDH+500	R	CTGACCTTGAGCTCTCCTTG
GAPDH+1000	F	GATTTCTCCTCCGGGTGATG
GAPDH+1000	R	CCAACTACCCATGACTCAGC
