# Supplementary material for: mRNA Cap Methyltransferase, RNMT-RAM, Promotes RNA Pol II-Dependent Transcription
Source: Cell Rep. 2018 May 2;23(5):1530–42. doi: 10.1016/j.celrep.2018.04.004 (PMC5946721; doi:10.1016/j.celrep.2018.04.004)
Supplement: Document S2. Article plus Supplemental Information [file mmc4.pdf]

Cell Reports

Article

mRNA Cap Methyltransferase, RNMT-RAM, Promotes RNA Pol II-Dependent Transcription

Graphical Abstract

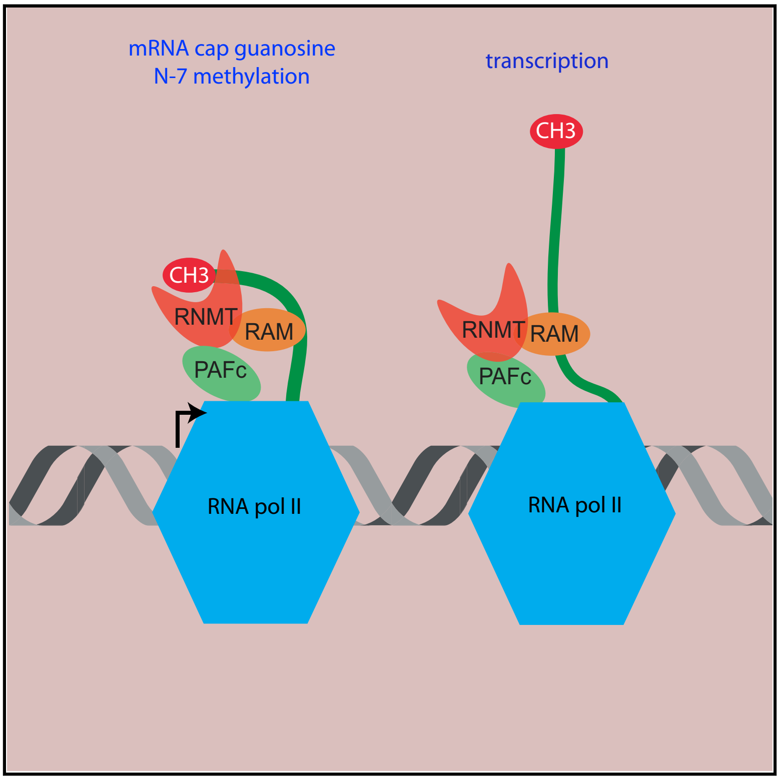

Authors

Dhaval Varshney, Olivia Lombardi, Gabriele Schweikert, Sianadh Dunn, Olga Suska, Victoria H. Cowling

Correspondence

v.h.cowling@dundee.ac.uk

In Brief

The mammalian mRNA cap methyltransferase, RNMT-RAM, prepares pre-mRNA for processing and translation and regulates expression of a subset of mRNAs. Here, Varshney et al. report that RNMT-RAM regulates transcription independent of mRNA cap methylation and translation. RNMT-RAM binds the full length of pre-mRNA and recruits proteins associated with transcription.

Highlights

- mRNA cap methyltransferase, RNMT-RAM, promotes RNA Pol II-dependent transcription
- RNMT-RAM-dependent transcription is independent of mRNA cap methylation
- RNMT-RAM binds to the entire length of pre-mRNA and to transcription-associated proteins
- Significant loss of RNA Pol II gene occupancy is observed on RNMT-RAM suppression

Data and Software Availability

GSE87767

Varshney et al., 2018, Cell Reports *23*, 1530–1542
May 1, 2018 © 2018 The Author(s).
https://doi.org/10.1016/j.celrep.2018.04.004

CellPress

OPEN ACCESS
CellPress

Cell Reports
Article

mRNA Cap Methyltransferase, RNMT-RAM, Promotes RNA Pol II-Dependent Transcription

Dhaval Varshney,[1,4] Olivia Lombardi,[1,5] Gabriele Schweikert,[2,3] Sianadh Dunn,[1,6] Olga Suska,[1] and Victoria H. Cowling[1,7,*]
[1]Centre for Gene Regulation and Expression, School of Life Sciences, University of Dundee, Dow Street, Dundee DD1 5EH, UK
[2]Division of Computational Biology, School of Life Sciences, University of Dundee, Dow Street, Dundee DD1 5EH, UK
[3]School of Informatics, University of Edinburgh, 10 Crichton Street, Edinburgh EH8 9AB, UK
[4]Present address: CRUK Cambridge Institute, Li Ka Shing Centre, Cambridge CB2 0RE, UK
[5]Present address: NDM Research Building, University of Oxford, Old Road Campus, Headington, Oxford OX3 7FZ, UK
[6]Present address: Astra Zeneca, Li Ka Shing Centre, CRUK Cambridge Institute, Robinson Way, Cambridge CB2 0RE, UK
[7]Lead Contact
*Correspondence: v.h.cowling@dundee.ac.uk
https://doi.org/10.1016/j.celrep.2018.04.004

SUMMARY

mRNA cap addition occurs early during RNA Pol II-dependent transcription, facilitating pre-mRNA processing and translation. We report that the mammalian mRNA cap methyltransferase, RNMT-RAM, promotes RNA Pol II transcription independent of mRNA capping and translation. In cells, sublethal suppression of RNMT-RAM reduces RNA Pol II occupancy, net mRNA synthesis, and pre-mRNA levels. Conversely, expression of RNMT-RAM increases transcription independent of cap methyltransferase activity. In isolated nuclei, recombinant RNMT-RAM stimulates transcriptional output; this requires the RAM RNA binding domain. RNMT-RAM interacts with nascent transcripts along their entire length and with transcription-associated factors including the RNA Pol II subunits SPT4, SPT6, and PAFc. Suppression of RNMT-RAM inhibits transcriptional markers including histone H2BK120 ubiquitination, H3K4 and H3K36 methylation, RNA Pol II CTD S5 and S2 phosphorylation, and PAFc recruitment. These findings suggest that multiple interactions among RNMT-RAM, RNA Pol II factors, and RNA along the transcription unit stimulate transcription.

INTRODUCTION

During the initial stages of eukaryotic pre-mRNA transcription, nascent transcripts are modified by mRNA cap addition (Furuichi, 2015; Shuman, 2015). The mRNA cap protects transcripts from 5′ exonucleases and recruits factors involved in splicing, nuclear export, and translation initiation (Gonatopoulos-Pournatzis and Cowling, 2014a; Topisirovic et al., 2011; Brannan et al., 2012). In addition to the cap protecting transcripts during synthesis, the capping enzymes can have roles in transcription. The *S. cerevisiae* cap methyltransferase, *ABD1*, is important for transcription of certain genes (Schroeder et al., 2004). Although a mechanism has not been defined, the transcriptional defects resulting from *ABD1* inactivation are rescued by the methyltransferase-dead enzyme, demonstrating independence from mRNA cap methylation. The *S. pombe* cap methyltransferase, PCM1, stimulates transcription by recruiting the elongation factor P-TEFb (Guiguen et al., 2007). A role for the mammalian mRNA cap methyltransferase in transcription has not been observed, although it is recruited to transcription initiation sites (Aregger and Cowling, 2013; Glover-Cutter et al., 2008).

The 7-methylguanosine cap is synthesized in a three-step process. A triphosphatase hydrolyses the 5′-phosphate of nascent transcripts and a guanylyltransferase adds guanosine monophosphate (GMP), linking guanosine to the first transcribed nucleotide via a 5′-to-5′-triphosphate linkage (Ramanathan et al., 2016). This guanosine cap is methylated at the N-7 position by a RNA cap methyltransferase. Although the capping enzymes have species-specific configurations, the triphosphatase, guanylyltransferase, and methyltransferase are all recruited to RNA polymerase II (RNA Pol II) and act co-transcriptionally on the nascent transcript (Buratowski, 2009; Perales and Bentley, 2009).

In different eukaryotic species, the mRNA cap methyltransferases have homologous catalytic domains, but their mode of recruitment to RNA Pol II differs. In *S. cerevisiae*, the cap methyltransferase ABD1 interacts directly with phosphorylated Serine 5 (phospho-S5) RNA Pol II C-terminal domain (CTD), whereas in *S. pombe*, the cap methyltransferase PCM1 is recruited in a complex with Cdk9/Pch1 (P-TEFb) (Pei et al., 2006; Schroeder et al., 2000). The mammalian cap methyltransferase, RNMT, has a N-terminal regulatory domain (residues 1–120) that regulates activity and mediates recruitment to transcription initiation sites, although a direct interaction with RNA Pol II has not been observed (Aregger and Cowling, 2013; Aregger et al., 2016; Pillutla et al., 1998). RNMT has an activating subunit, RAM, which alters the dynamics of key active site residues, improving the environment for methyl donor binding (Gonatopoulos-Pournatzis et al., 2011; Varshney et al., 2016). RAM also has a high-affinity RNA binding domain, the biochemical function of which is unclear; it is required for cell viability but does not increase RNMT methyltransferase activity (Gonatopoulos-Pournatzis et al., 2011; Gonatopoulos-Pournatzis and Cowling, 2014b). The RAM RNA binding domain may increase the recruitment of specific transcripts to the complex or have a function

 © 2018 The Author(s).
This is an open access article under the CC BY license (http://creativecommons.org/licenses/by/4.0/).

independent of mRNA cap methylation. In embryonic stem cells, the expression of a subset of pluripotency-associated transcripts depends on RAM, indicating a role for RAM in transcription or RNA stability (Grasso et al., 2016).

Here we report that RNMT-RAM functions independently of mRNA cap methylation to promote RNA Pol II-dependent transcription. Sublethal suppression of RNMT-RAM expression results in massively reduced RNA Pol II occupancy, reduced net mRNA synthesis, and reduced pre-mRNA levels. Increasing RNMT-RAM expression in cells increases transcription in a methyltransferase-independent manner. Furthermore, recombinant RNMT-RAM stimulates transcription in isolated nuclei, confirming that this mechanism is independent of mRNA capping, mRNA translation, and mRNA stability. RNMT-RAM associates with pre-mRNA along its entire length via interaction with RAM and interacts with several transcription-associated complexes. We propose that the human cap methyltransferase complex promotes transcription by RNA Pol II via multiple RNA and protein contacts along the transcription unit.

RESULTS

RNMT-RAM Promotes RNA Pol II-Dependent Transcription

To investigate the cellular role of the mRNA cap methyltransferase, RNMT-RAM, HeLa cells were transfected with two RAM small interfering RNAs (siRNAs), which reduce expression of RNMT and RAM (Figure 1A) (Gonatopoulos-Pournatzis et al., 2011). To focus these experiments on the role of RNMT-RAM, it was important to use a sublethal dose of RAM siRNA. Under the conditions used here, transfection of RAM siRNA for 48 hr did not result in toxicity; there was no reduction in cell number, no apoptotic morphology, and no induction of apoptosis as detected by PARP cleavage (Figure S1). To investigate cellular transcription, cells were incubated with ^{3}H-uridine for 30 min, which is converted to ^{3}H-UTP and incorporated into nascent transcripts. mRNA was enriched by oligonucleotide (oligo)-dT affinity, and ^{3}H-uridine incorporation was determined. Because the median half-life of mRNA is $\sim$10 hr, a 30 min uridine pulse predominantly measures transcription (Yang et al., 2003). Net mRNA synthesis was significantly reduced following RNMT-RAM depletion (Figure 1A). In a similar experiment, while RAM was depleted by RAM siRNA transfection, HA-tagged RNMT (HA-RNMT) was induced from a doxycycline-regulated promoter (Figure 1B). HA-RNMT expressed in the absence of RAM was unable to increase net mRNA synthesis. Conversely, elevated expression of HA-RNMT and Fg-RAM (in the absence of siRNA transfections) increased net mRNA synthesis (Figure 1C). Furthermore, co-expression of methyltransferase-dead (MTD) HA-RNMT and Fg-RAM increased net mRNA synthesis (Gonatopoulos-Pournatzis et al., 2011). Therefore, RNMT-RAM promotes transcription independent of its role in mRNA cap methylation.

To investigate whether RNMT-RAM has a direct impact on transcription, nuclear run-on assays were performed. Nuclear run-on measures nascent transcription from engaged polymerase in isolated nuclei, independent of mRNA capping, translation, and stability (Groudine et al., 1981). In these assays, nuclei prepared from log-phase HeLa cells were supplemented with nucleotide triphosphates (NTPs), BrUTP (5-bromouridine-triphosphate), and RNase inhibitors, and transcription was allowed to progress (Roberts et al., 2015). Bromouridine (Br-U) is incorporated into nascent transcripts, which are immunoprecipitated and used as a substrate for RT-PCR. Addition of recombinant RNMT-RAM resulted in up to a 1.5- to 2-fold increase in TSS (transcription start site) and gene body-associated transcription of multiple genes (Figures 1D, S2A, and S2B). For these assays, full-length human RNMT and RAM 1–90 were used; full-length recombinant RAM 1–118 is unstable (Gonatopoulos-Pournatzis et al., 2011). Thus, RNMT-RAM directly promotes transcription, increasing the output of engaged RNA Pol II, independent of mRNA cap methylation. On supplying RNMT and RAM alone to isolated nuclei, RAM 1–90 alone could stimulate transcription, whereas RAM 1–45, a mutant in which the RNA binding domain is deleted, could not (Figure S2A).

To further evaluate the mechanism by which RNMT-RAM influences transcription, we required the identity of the genes most affected by its inhibition. RNA sequencing was performed on HeLa cells following RAM depletion for 36 hr in 5 biological replicates (Figures S2C–S2E). Despite the overall transcriptional repression, RNA sequencing (RNA-seq) analysis revealed that different transcript populations respond differentially to RAM depletion. For instance, transcripts within the most repressed decile and the least repressed decile exhibit a more than 2.5-fold difference in reduction in response to RAM depletion (Figure S2E; Data S1). This indicates that there may be gene specificity in transcriptional regulation by RAM. The expression of a panel of mRNAs was validated by RT-PCR (Figures S2E and S2F). Transcripts coding for the RNA binding protein hnRNPH1, phosphodiesterases 3A and 4D, transcription factor FOXP2, and two non-coding RNAs MALAT1 and NEAT1, as well as GPC5, GAPDH, GSG2, and RNMT, were selected for further analysis. The transcripts most reduced in response to RAM depletion may be transcribed at a lower rate or have reduced stability. To assess the impact of RAM depletion on pre-mRNA levels, RT-PCR was performed on RAM-dependent transcripts using intronic primers. Following RAM depletion, hnRNPH1, PDE3A, PDE4D, and FOXP2 pre-mRNA levels reduced equivalently to mature mRNA levels, consistent with RNMT-RAM controlling transcription (Figure 1E). mRNA stability was assessed following transcriptional inhibition with actinomycin D. The decay of hnRNPH1, PDE4D, FOXP2, and MALAT1 mRNA was unchanged following RNMT-RAM depletion (Figure S3).

RAM Depletion Results in Reduced RNA Pol II Occupancy

To define the role of RAM in transcription, RNA Pol II occupancy on the genome was investigated using chromatin immunoprecipitation (ChIP). Following transfection with RAM siRNA or non-targeting control for 36 hr, RNA Pol II ChIP was performed on three biological replicates that exhibited a high correlation genome wide and at an individual gene level (Figures S4A and S4B). In genome-wide analysis, RAM depletion was observed to result in a significant reduction in RNA Pol II occupancy at the TSS (Figure 2A) and within the gene body (Figures 2B and S5). In single-gene analysis, 3,400 genes exhibited a significant change in RNA Pol II binding over the annotated locus

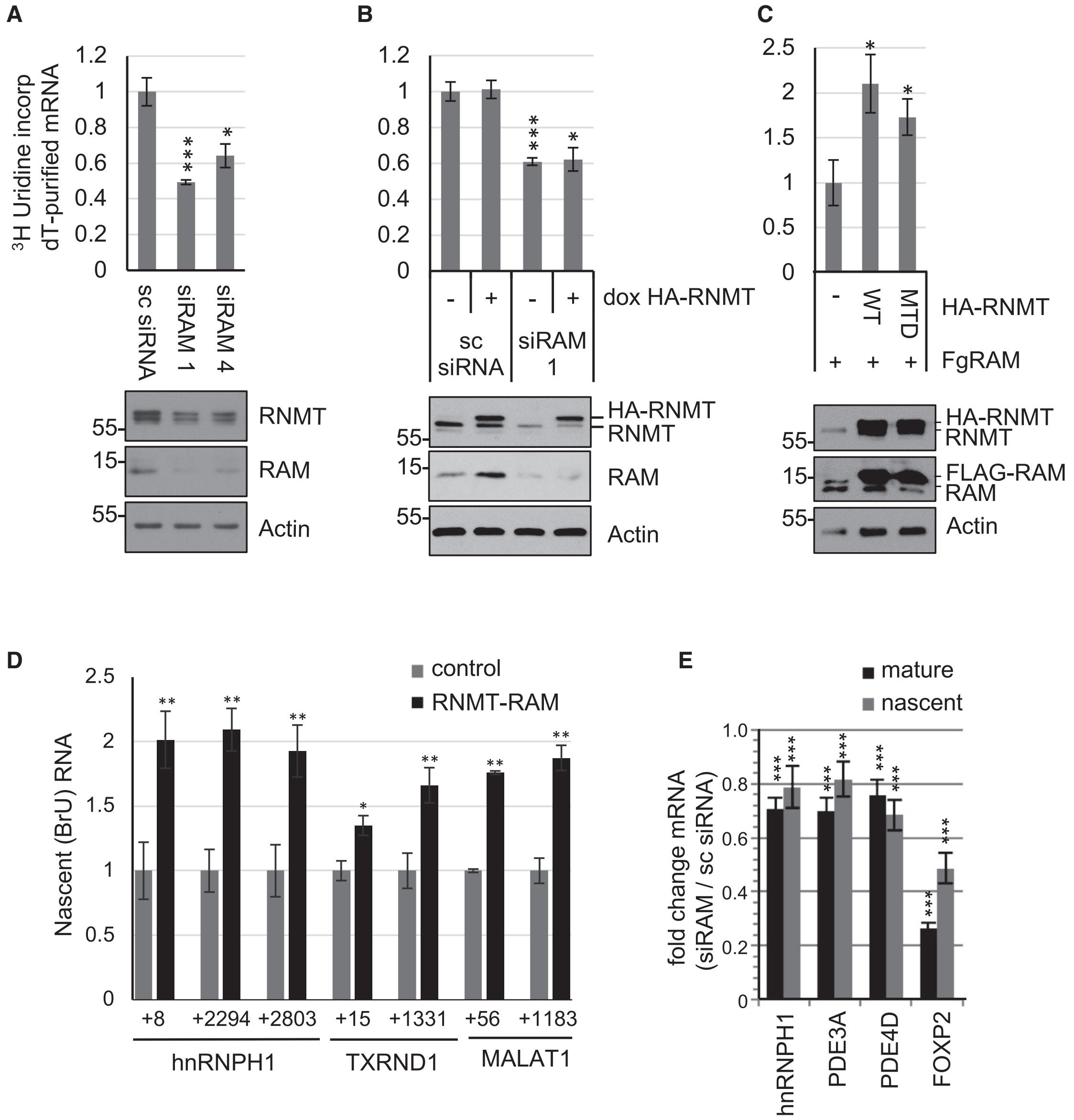

Figure 1. RNMT-RAM Regulates Transcription Independent of mRNA Cap Methylation

(A–C) HeLa cells incubated with 60 μM ^{3}H-uridine for 30 min. Transcripts were poly(A) selected. Relative ^{3}H-uridine incorporation was normalized to poly(A) RNA (n = 3). (A) Cells transfected with RAM siRNAs or non-targeting control (sc siRNA) for 36 hr. (B) Cells transfected with RAM siRNA or sc siRNA for 36 hr, and HA-RNMT was induced with doxycycline for 12 hr. (C) Cells transfected with pcDNA5 Fg-RAM and pcDNA5 HA-RNMT wild-type (WT), methyltransferase-dead (MTD), or vector control for 48 hr. Representative western blots are shown.

(D) HeLa nuclei incubated with NTPs, BrUTP, and recombinant RNMT (FL)-RAM (1–90) for 20 min. Br-RNA was purified and used as a substrate for RT-PCR. Primers are indicated (n = 4).

(E) HeLa cells transfected with RAM siRNAs or sc siRNA for 36 hr. Levels of mature and pre-mRNA were determined by RT-PCR relative to sc siRNA control (n = 4). For charts, average and SD are indicated. Student's t test was performed. $^*p < 0.05$, $^{**}p < 0.01$, $^{***}p < 0.005$.

and 2 kb flanking regions, of which 89% exhibited reduced RNA Pol II occupancy (Figure 2C; Data S1). This was also visible at the single genes investigated previously (Figure S6). The RNMT locus exhibited an increase in RNA Pol II occupancy on RNMT-RAM suppression, indicating a feedback response (Figure S6).

An alternative approach to investigating RNA Pol II occupancy is to identify RNA Pol II peaks throughout the genome, i.e., regions where the RNA Pol II ChIP reads are significantly enriched relative to the input sample. Peak calling identified 9,464 consensus peaks in control cells, whereas following RAM depletion, only 1,323 peaks were identified, an 86% reduction (Figures 2D and S7). The RNA Pol II peaks remaining following RAM depletion were predominately (1,309 peaks) in the same location as in control cells. For individual RNA Pol II peaks identified in

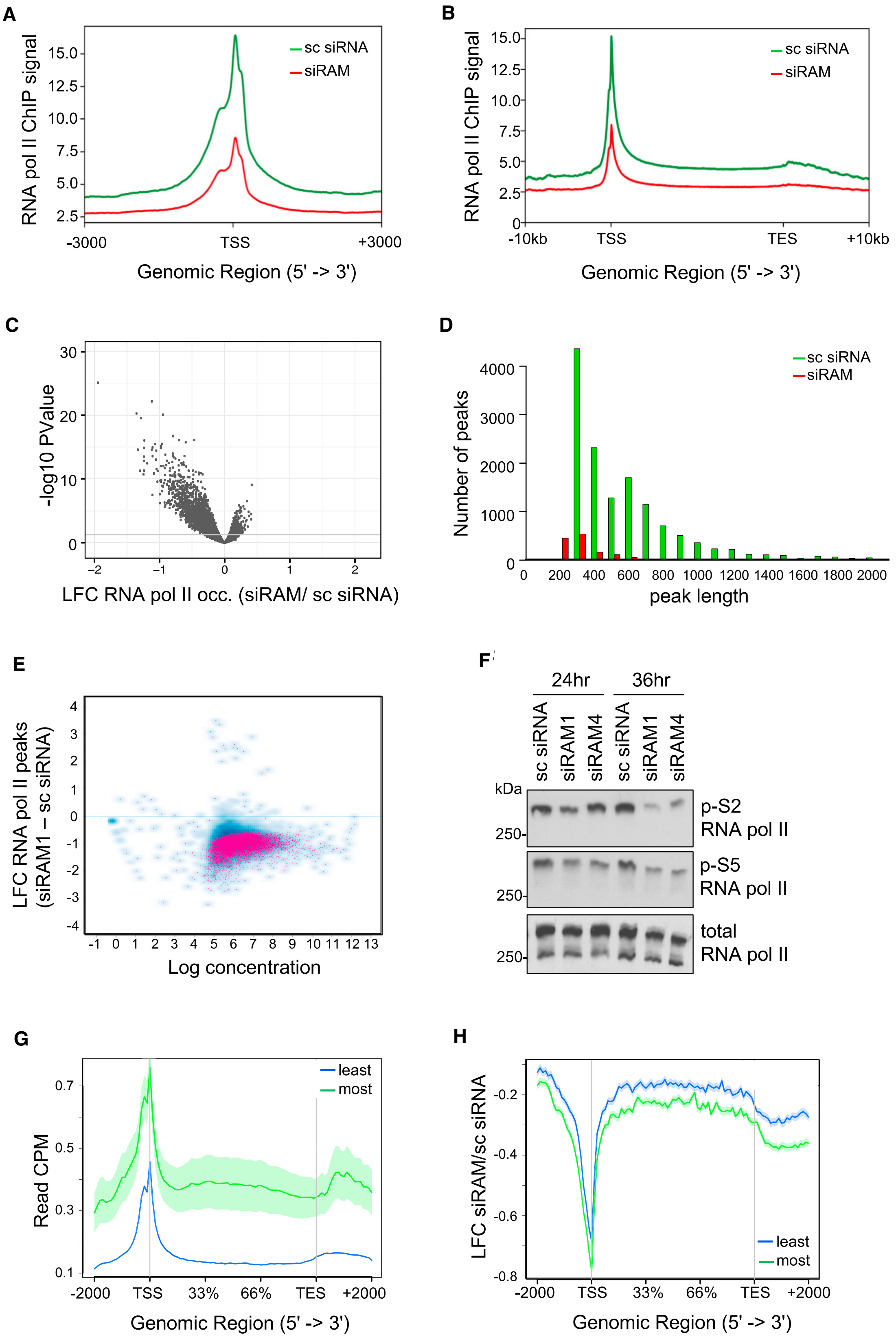

(legend on next page)

control cells, a significant reduction in read intensity was observed in response to RAM depletion across a range of peak read densities (Figure 2E). Consistent with a reduction in RNA Pol II at the TSS and gene body, suppression of RAM expression resulted in a reduction in phospho-S5 and phospho-S2 RNA Pol II (Figure 2F).

RNMT-RAM suppression not only had a major impact on genome-wide RNA Pol II loading (Figure 2A–2E) but also elicited a transcript-specific response (Figure S2E). Therefore, we investigated which properties differentiated genes with different dependencies on RNMT-RAM. In control cells, from the 11,917 transcripts detected by RNA-seq (Figure S2E), the 4,007 transcripts most repressed in response to RNMT-RAM suppression had significantly more RNA Pol II loaded throughout the associated gene in comparison to the 4,059 least repressed transcripts, indicating that these two genes sets have distinct properties (Figure 2G). When RNMT-RAM was suppressed, as expected, RNA Pol II was depleted more at the most repressed genes than at the least repressed genes (Figure 2H).

RAM Is Required for RNMT Interaction with RNA

The impact of RNMT-RAM on RNA Pol II occupancy and transcription suggested a potential functional interaction with transcribing polymerase. To investigate the mechanism of RNMT-RAM-dependent transcription, the RNAs to which RNMT-RAM binds (Figures 3 and 4) and RNMT-RAM-interacting proteins (Figure 5) were identified. Cellular protein-RNA complexes were captured by CLIP (crosslinking immunoprecipitation) (Huppertz et al., 2014; Moore et al., 2014). HA-RNMT was introduced into HeLa cells by retroviral infection, resulting in expression equivalent to endogenous RNMT (Figure S8A). RNA was cross-linked to proteins by UV radiation; RNase-treated and HA-RNMT-RAM-RNA complexes were immunoprecipitated using anti-hemagglutinin (HA) antibodies. Following ^{32}P RNA labeling, RNMT-RNA and RAM-RNA complexes were visualized in SDS-PAGE as smears, migrating more slowly than RNMT (66 kDa) and RAM (14 kDa) (Figure 3A). Protein-RNA bands were only detected following mild RNase treatment, indicating release of RNMT-RAM-RNA from masking and/or insoluble complexes, and were diminished at high RNase concentrations, confirming their derivation from RNA (Figure 3A). The 50 kDa band observed is likely to be non-specifically labeled immunoglobulin G (IgG) heavy chain.

The dependency of RNMT-RNA binding on RAM was investigated. RNA binding to the RNMT lobe deletion mutant (Δ416–Δ456) was below the limit of detection (Figure 3B). This mutant is defective for RAM binding, which indicates that RAM is necessary for transcripts to bind efficiently to RNMT in cells (Varshney et al., 2016). In mouse embryonic stem cells (mESCs), RNMT and RAM expression is uncoupled, providing a system to study the action of RNMT independent of RAM (Grasso et al., 2016). Upon RAM depletion in mESCs, RNA binding to wild-type RNMT was severely diminished (Figure 3C). The RNA binding domain of RAM was previously mapped *in vitro* to the asparagine- and arginine-rich (NR-rich) region (amino acids 56–91), and RAM 1–55 binds to RNMT (Gonatopoulos-Pournatzis et al., 2011). To map the regions of RAM required to bind to RNA in cells, GFP-CLIP assays were performed in which a series of RAM-GFP deletion mutants were purified from HeLa cells (Figure 3D). Although the PY nuclear localization motifs at amino acids 98 and 114 are absent from RAM 1–90, all RAM-GFP proteins in this study are predominantly nuclear, probably due to nuclear bias of GFP (Gonatopoulos-Pournatzis and Cowling, 2014b). As expected, wild-type RAM-GFP (1–118) bound to RNA. However, all other RAM-GFP deletion mutants were defective for RNA binding. Therefore, full-length RAM is required for efficient RNA binding in cells.

RNMT-RAM Binds the Entire Length of Pre-mRNA

Transcripts bound to HA-RNMT-RAM complexes were isolated from HeLa cells using CLIP and RNA fragments sequenced as in Huppertz et al. (2014) (Figure S8B). The quantity of RNMT-RAM-bound RNA fragments identified per transcript from three independent replicates exhibited a good correlation (Figure S8C). The average RNMT CLIP reads per kilobase per million mapped reads (RPKM) from three replicates was compared to input RPKM (Figure 4A). This revealed a positive correlation between transcript expression level and RNMT binding (Pearson's correlation R = 0.83). However, not all abundantly expressed transcripts were RNMT bound (population adjacent to the x axis), and a subset of RNMT-bound transcripts was below the limit of detection in the input sample (population adjacent to the y axis).

Genome coverage analysis of the 727,624 uniquely aligned RNMT-RAM CLIP reads pooled from the three replicates revealed that most aligned to intronic regions of pre-mRNA, as opposed to the 5′ localization expected of a 5′ cap methyltransferase (Figure 4B). Long non-coding RNAs (lncRNAs) were also bound by RNMT-RAM, including some highly enriched ones, e.g., MALAT1 and NEAT1 (Data S1). Because introns represent a large proportion of the annotated human genome, RNMT-RAM CLIP reads per kilobase of mRNA features were calculated (Figure 4C). RNMT-RAM CLIP reads were evenly distributed

Figure 2. RNMT-RAM Regulates RNA Pol II Occupancy

(A and B) RNA Pol II ChIP-seq reads over annotated transcription start sites (A) and annotated genes (B) within the hg38 human assembly, 36 hr post-transfection with siRAM (red line) or sc siRNA (green line). Signal was normalized to inputs.

(C) Volcano plot of $\log_2$ fold change in RNA Pol II ChIP-seq reads uniquely aligned to gene locus ± 2 kb following RAM siRNA transfection and $\log_{10}$ p value. Horizontal line, p value of 0.05.

(D) Number of RNA Pol II ChIP peaks called using MACS2 plotted against peak length for sc siRNA- and siRAM-transfected cells.

(E) Scatterplot of $\log_2$ fold change of RNA Pol II peaks following RAM depletion. False discovery rate (FDR) < 0.05 is highlighted.

(F) HeLa cells transfected with two RAM siRNAs or sc siRNA for 24 and 36 hr. Western blot analysis is shown.

(G) RNA Pol II ChIP-seq reads from control siRNA-transfected cells. Genes for transcripts were categorized as most reduced and least reduced in RNA-seq following RAM depletion (Figure S2E).

(H) Log fold change in RNA Pol II ChIP-seq reads following RAM knockdown over gene sets plotted in (G).

Data were pooled from three biological replicates for (A)–(E). TSS, transcription start site; TES, transcription end site.

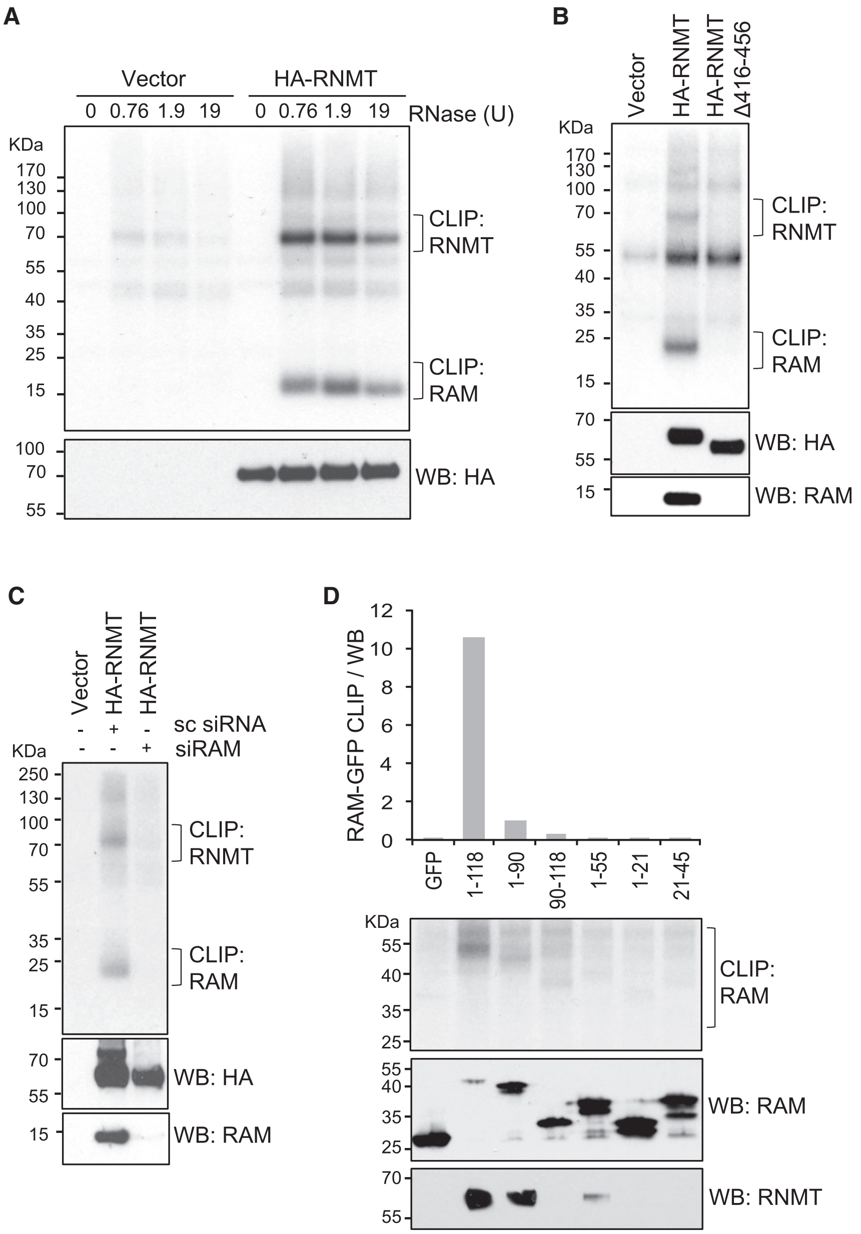

Figure 3. RAM Is Required for RNMT-RNA Interaction

(A) Anti-HA antibody CLIP (crosslinking IP) performed on HeLa cells expressing HA-RNMT or control. IP was performed with or without prior incubation with RNase titration. ^{32}P-labeled transcripts were visualized by autoradiography (0.38 U RNase was used in subsequent assays).

(B) Anti-HA antibody CLIP performed on HeLa cells expressing HA-RNMT WT, Δ416–Δ456, and vector control.

(C) mESC expressing HA-RNMT transfected for 72 hr with RAM siRNA or non-targeting control. Anti-HA antibody CLIP was performed.

(D) Anti-GFP CLIP performed on HeLa cells transfected with pcDNA5 GFP and GFP-RAM constructs. Quantification of the RAM CLIP signal was normalized to the RAM western blot signal.

CLIPs and western blots were performed on the same membrane.

between exons and introns but had increased enrichment over the UTRs. A correlation between UTR length and RNMT binding was not observed (Figure S9A). Because RNMT-RAM binds to transcript introns (Figures 4B and 4C) and requires RNase treatment to be resolved on SDS-PAGE (Figure 3A), it is likely that the complex binds to pre-mRNA during transcription.

Although transcripts from 5,436 genes were identified as binding to RNMT (average log counts per million [logCPM] > 5), for further mechanistic analysis, the 684 transcripts found in the top quartile of all three RNMT-RAM CLIP replicates were designated as RNMT-enriched transcripts (Figure S9B; Data S1). Analysis of RNMT CLIP read distribution over these RNMT-enriched transcripts confirmed that the RNMT-RAM complex binds the entire length of the nascent transcripts (Figure 4D).

RNMT-RAM Interacts with the Transcription-Associated Complexes

Because RNMT-RAM binds to the full length of pre-mRNA and promotes transcription, we investigated whether it binds to transcription-associated proteins. SILAC (stable isotope labeling of amino acids in cell culture) media were used to differentially label cellular proteins, allowing comparative quantitation. HA-RNMT was immunoprecipitated from extracts of cells grown in R6K4 medium, and a mock IgG immunoprecipitation (IP) was performed on extracts of cells grown in R0K0 medium (Figure 5A). Mass spectrometry was used to identify proteins enriched in the HA-RNMT IP compared to the IgG IP (Figure 5B; Data S2). HA-RNMT was found to interact with components of transcription-associated complexes, including three RNA Pol II subunits, POLR2G, POLR2H, and POL2L; transcription elongation proteins SPT4, SPT6, and ELP3; and members of the PAF complex, PAF1, CTR9, CDC73, and LEO1.

Although all previously identified RNMT-RAM-interacting proteins may have roles in RNMT-RAM-dependent transcription, we restricted further analysis to the PAF complex (PAFc). PAFc binds directly to RNA Pol II across the gene and is involved in transcription initiation and elongation by interacting with transcriptional regulators and promoting many aspects of the transcription cycle (Jaehning, 2010; Kim et al., 2010; Wade et al., 1996; Yu et al., 2015). The mechanistic relationship between RNMT-RAM and PAFc was further investigated. Endogenous CTR9 was identified in RNMT and RAM IPs (Figure 5C), and endogenous RNMT was identified in CTR9 IPs (Figure 5D). Endogenous LEO was also identified in RNMT IPs (Figure 5E).

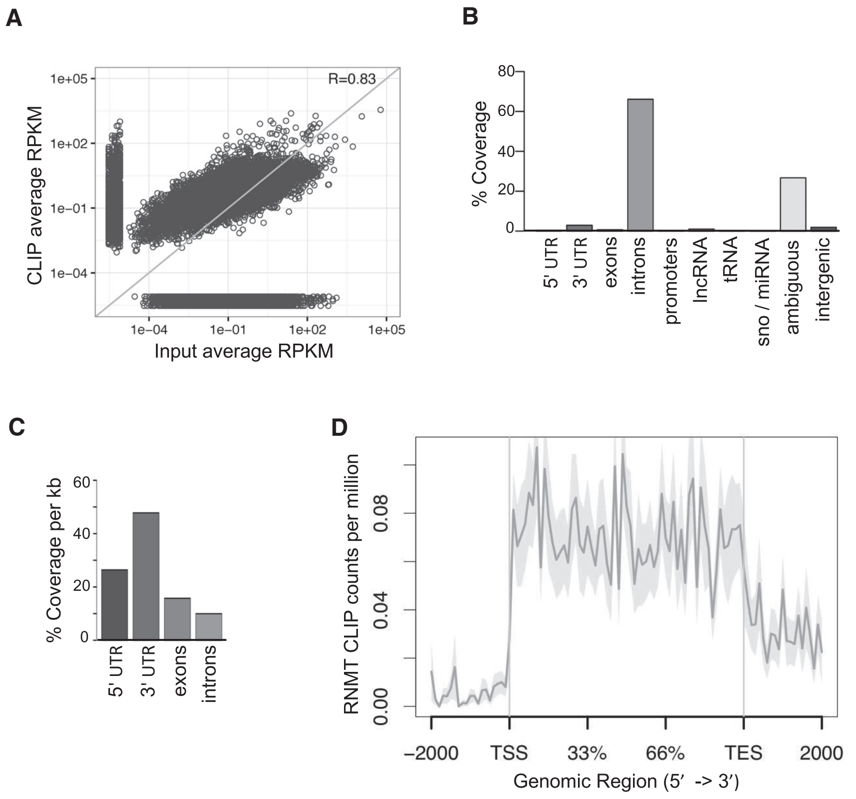

Figure 4. RNMT-RAM Binds throughout mRNA

(A) Scatterplot of RNMT CLIP reads and inputs expressed as reads per kilobase per million mapped reads (RPKM) for each annotated gene locus.

(B) Percentage of genome coverage for CLIP reads calculated over annotated regions of the hg38 genome assembly. Reads aligning to unannotated genomic regions were assigned as intergenic. Overlapping annotation was assigned as ambiguous.

(C) Genome coverage calculated per kilobase of annotated mRNA features. Ambiguous is excluded.

(D) Average profile from coverage splines of HA-RNMT CLIP reads over 684 RNMT-enriched target gene bodies. Shaded area represents SEM for each bin.

Multiple members of the yeast PAFc bind RNA *in vitro* and in cells (Battaglia et al., 2017; Dermody and Buratowski, 2010). The RNA dependency of the RNMT and PAFc subunit interaction was investigated. RNase A treatment did not disrupt the RNMT-CTR9 interaction, indicating that it is RNA independent (Figure 5C). CTR9 interacted with the RNMT catalytic domain (Figure 5F). The impact of RNMT-RAM on PAFc recruitment to genes was assessed by investigating the occupancy of the CTR9 subunit. CTR9 recruitment to three RAM-responsive genes, hnRNPH1, FOXP2, and PDE3A, and one control gene, GAPDH, was investigated by ChIP (Figure 5G). CTR9 recruitment to hnRNPH1, FOXP2, and PDE3A genes was reduced following RAM siRNA transfection. A smaller reduction in CTR9 recruitment to the GAPDH gene was observed.

Consistent with its influence on PAFc recruitment, suppression of RAM expression resulted in suppression of hallmarks of PAFc function. PAFc co-operates with the Mediator complex to stimulate the mono-ubiquitination of histone H2B at lysine 120 (H2Bub), which is critical for histone H3 lysine 4 trimethylation (H3K4me3) (Shilatifard, 2008; Yao et al., 2015). PAFc also stimulates RNA Pol II CTD S2 phosphorylation (phospho-S2); this recruits Set2, which in turn catalyzes histone H3 lysine 36 trimethylation (H3K36me3) (Schwartz et al., 2003; Yu et al., 2015). RAM depletion resulted in suppression of H2Bub, H3K4me3, and H3K36me3 (Figure 5H) and suppression of RNA Pol II CTD phospho-S5 and phospho-S2 (Figure 2F). RNMT-RAM interaction with POLR2G, POLR2H, POL2L, SPT4, SPT6, and ELP3 is also likely to affect these markers of transcription.

RNMT-RAM-RNA Interaction Is Required for RNA Pol II Occupancy

Because RNMT-RAM binds across the length of pre-mRNA, binds to transcriptional regulators, and promotes transcription, the relationship between RNMT-RAM transcript binding and RNA Pol II occupancy was investigated. Genome-wide, RNMT-RAM transcript binding correlated positively with RNA Pol II occupancy over associated gene loci (Figure 6A; genes products most enriched for RNMT binding are highlighted in red). In control cells, genes encoding the RNMT-enriched transcripts exhibited significantly higher levels of RNA Pol II loading than the remaining genes (mean logCPM RNMT enriched = 7.58; mean logCPM unenriched = 5.5; $p < 2.2e-16$) (Figures 6B and 6C). Upon RAM depletion, the genes encoding RNMT-enriched transcripts exhibited a greater fold reduction in RNA Pol II occupancy than the remaining genes (Figure 6D). This was particularly evident at the TSS, where the mean log fold change for RNMT-enriched genes was −0.80 and for unenriched genes was −0.47 ($p < 0.0001$). Furthermore, the RNMT-enriched genes exhibited the largest decrease in RNA Pol II peak intensity (Figure 6E; transcripts most enriched for RNMT binding are highlighted in red). Most (99%) genes with a significant response to RAM depletion exhibited a reduction in TSS-associated RNA Pol II (Figure 6F).

DISCUSSION

Here, we report that RNMT-RAM promotes RNA Pol II-dependent transcription. The impact of RNMT-RAM on transcription is direct and independent of mRNA capping, mRNA stability, and mRNA translation; expression of MTD RNMT-RAM promotes net mRNA synthesis in cells, and RNMT-RAM promotes the output of engaged RNA Pol II in isolated nuclei (Figure 1). RNMT-RAM binds to pre-mRNA during transcription along the length of the transcript, in positive correlation with RNA Pol II gene occupancy (Figures 3, 4, and 6). When RNMT-RAM is suppressed, there is a substantial loss of RNA Pol II from chromatin; 86% of RNA Pol II

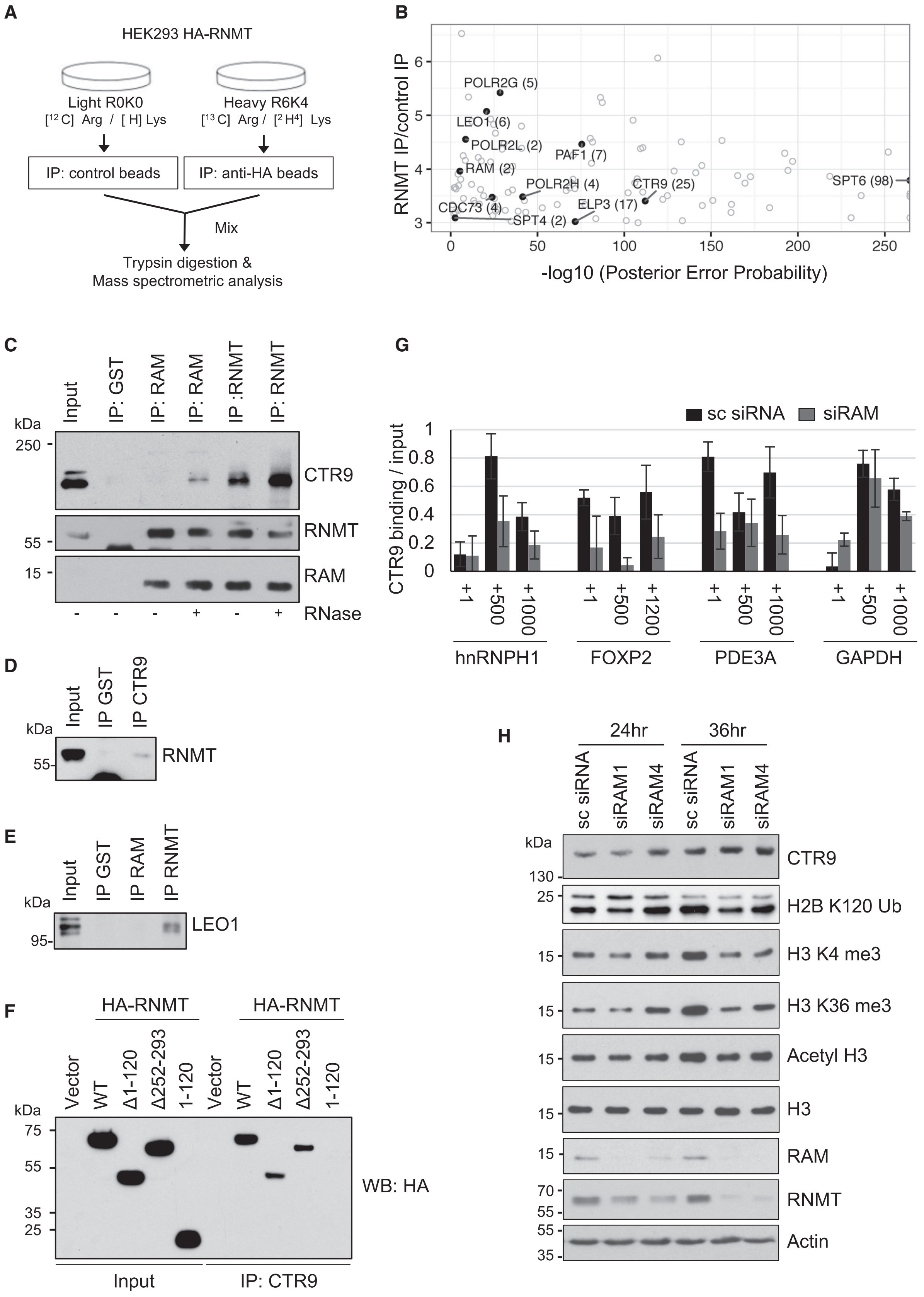

(legend on next page)

OPEN ACCESS
CellPress

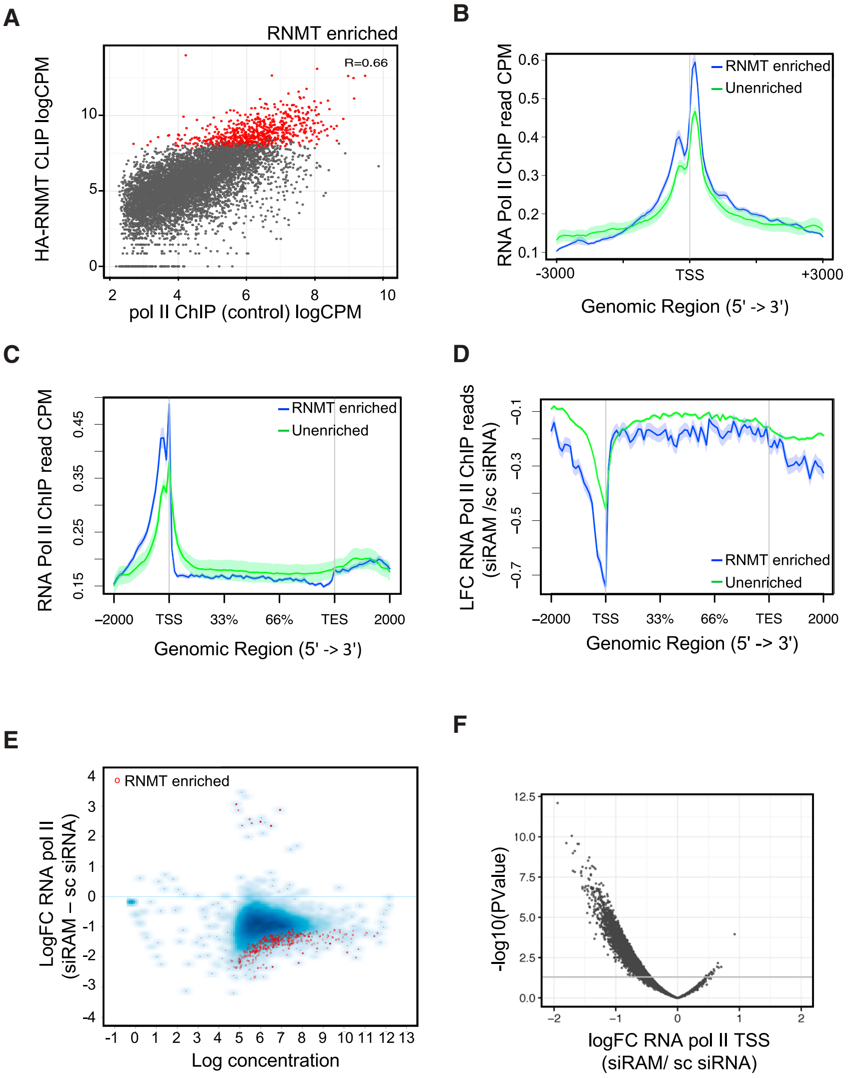

Figure 6. RAM Depletion Affects RNA Pol II Occupancy on Genes Coding RNMT-Enriched Transcripts

(A) Scatterplot of RNA Pol II ChIP reads in control cells and HA-RNMT CLIP reads for annotated loci. R value, Pearson's correlation. Genes coding 684 RNMT-enriched CLIP transcripts are in red.

(B and C) RNA Pol II ChIP read distribution over annotated TSS (B) and gene body (C) for RNMT-enriched genes (blue line) and remaining annotated loci (green line). Signal is normalized to inputs. Loci with no signal are not plotted. Shaded area represents SEM for each bin.

(D) Log_2 fold change in RNA Pol II ChIP reads in HeLa cells following RAM siRNA transfection compared to sc siRNA. 684 genes enriched in HA-RNMT CLIP, blue line; remaining loci, green line. Data are pooled from three independent biological replicates. Shaded area, SEM per bin.

(E) Scatterplot of $\log_2$ fold change of RNA Pol II peaks following RAM depletion. RNMT-enriched genes are indicated.

(F) Volcano plot of $\log_2$ fold change in RNA Pol II ChIP reads uniquely aligned ±1 kb TSS following RAM siRNA transfection and $-\log_{10}$ p value. Horizontal line, p value of 0.05.

peaks reduce to below the threshold of detection (Figure 2). The impact of RNMT-RAM on transcription is most apparent on genes to which RNMT-RAM binds the RNA products (Figure 6). We emphasize that in this study, we established protocols for sublethal depletion of RNMT-RAM in cells, allowing the impact of RNMT-RAM to be studied independently of toxicity (Figure S1).

How does RNMT-RAM affect transcription? The activating subunit, RAM, has a key role. Previously, both the RNMT-activating domain (1–55) and the RNA binding domain (55–90) were demonstrated to be important for cell proliferation (Gonatopoulos-Pournatzis and Cowling, 2014b). However, the mechanistic role of the RNA binding domain was unclear. On analysis of RNMT-RAM-bound transcripts, we did not identify a specific RNA motif to which the complex has enhanced affinity; rather, RAM was critical for RNMT to be recruited to all transcripts along their entire length (Figures 3 and 4). As discussed, RNMT-RAM recruitment to transcripts positively correlates with RNA Pol II loading on genes, consistent with RNMT-RAM being recruited to RNA Pol II; the more nascent transcript emerging, the more RNA for RNMT-RAM to bind (Aregger and Cowling, 2013; Glover-Cutter et al., 2008). What was unexpected was that suppression of RNMT-RAM expression resulted in repression of RNA Pol II occupancy on genes (Figure 2), and the extent of this effect correlated with RNMT-RAM transcript binding (Figure 6).

Figure 5. RNMT-RAM Interacts with Transcription-Associated Complexes

(A) Identification of HA-RNMT-interacting proteins using SILAC-based mass spectrometry.

(B) Fold enrichment of proteins in HA-RNMT IP/IgG IP plotted against the $-\log_{10}$ posterior error probability score. Transcription-associated proteins are highlighted (with the number of peptides identified in parentheses). Three-fold enrichment over control cutoff.

(C–E) HeLa cell extracts subject to IP-western blot analysis for CTR9 (C), RNMT (D), and LEO1 (E). Where indicated, IPs were incubated with or without RNase (C). Anti-glutathione S-transferase (GST) IP, negative control.

(F) HeLa cells transfected with pcDNA5 HA-RNMT WT and mutants or vector control for 48 hr. Extracts were subject to CTR9 IP and HA western blot analysis.

(G) HeLa cells transfected with RAM siRNA or non-targeting control for 36 hr. CTR9 ChIP was performed. Average and SEM are presented for the PCR signal relative to input (background subtracted) ($n \geq 3$).

(H) HeLa cells transfected with two RAM siRNAs or sc siRNA for 24 and 36 hr. Western blot analysis is shown.

One feature of the impact of RNMT-RAM on RNA Pol II distribution is that it reduces RNA Pol II at the TSS and the gene body (Figures 2 and 6). RNA Pol II at the TSS can be highly unstable, undergoing rounds of premature transcription termination (Ehrensberger et al., 2013; Wagschal et al., 2012). Findings demonstrated that RNA Pol II at the TSS, rather than being stably paused, can be rapidly turning over, and induced transcription occurs when the dynamic RNA Pol II is stabilized (Krebs et al., 2017; Nilson et al., 2017). Nuclear run-on experiments demonstrated that RNMT-RAM increases RNA Pol II output throughout the gene. Our data are consistent with RNMT-RAM promoting RNA Pol II stabilization following initiation, thus increasing the peak proximal to the TSS and promoting RNA Pol II output.

Which factors mediate RNMT-RAM-dependent transcription? A study in *S. cerevisiae* reported that many RNA Pol II-associated factors, including PAFc, crosslink to nascent RNA (Battaglia et al., 2017). The authors propose that interaction with nascent RNA contributes to elongation factor recruitment to transcribing RNA Pol II. The multiple contacts between RNMT-RAM, transcription-associated factors (RNA Pol II subunits SPT4, SPT6, and the PAFc), and pre-mRNA are likely to stabilize their interaction and retain these complexes on the transcript during synthesis (Figures 3, 4, and 5). We demonstrated that RNMT-RAM suppression results in dissociation of PAFc from its target loci (Figure 5). Therefore, both RNA Pol II and Ctr9 recruitment are reduced by inhibition of RNMT-RAM. Because PAF controls RNA Pol II action and, conversely, RNA Pol II has a PAF recruitment surface, it is not possible to determine which is the primary RNMT-RAM-controlled factor; it may be a combination of both.

Previous studies described a checkpoint model for coupling mRNA capping with transcription (Orphanides and Reinberg, 2002; Saldi et al., 2016). Several such mechanisms have been observed, including those that operate in global and gene-specific manners. The first step in cap formation, addition of inverted guanosine to the nascent transcript, is a necessary checkpoint to protect the pre-mRNA from degradation during synthesis, probably in all eukaryotes (Brannan et al., 2012; Furuichi et al., 1977). In addition, the capping enzymes have been observed to regulate transcription independent of enzymatic activity; such mechanisms identified to date operate in gene-specific manners. Because the relationship between the capping enzymes and the transcriptional machinery is fairly distinct in different eukaryotes, it is perhaps unsurprising that these enzymes influence transcription in species-specific mechanisms. In yeast species, the mRNA cap guanylyltransferases and methyltransferases interact with transcriptional regulators and have roles in promoting transcription (Guiguen et al., 2007; Kim et al., 2004; Schroeder et al., 2004). In mammals, the capping enzyme (RNGTT) (triphosphatase-guanylyltransferase) counteracts transcriptional repression by the negative elongation factor (NELF) and promotes formation of transcriptional R loops (Kaneko et al., 2007; Mandal et al., 2004). The role of the human mRNA cap methyltransferase in transcription had not been addressed previously.

The RNMT-RAM checkpoint discovered here is a mechanism to regulate transcription, which results in gene-specific changes in RNA Pol II occupancy and output. This is physiologically important in systems in which RNMT-RAM is regulated. For example, in embryonic stem cells, RNMT-RAM influences the expression of pluripotency-associated genes (Grasso et al., 2016). Repression of RAM is required for repression of these genes during differentiation.

In summary, we present a revised understanding of RNMT-RAM function. Because mRNAs are capped and depend on RNMT-RAM for translation, it is likely that a transient interaction with the cap methyltransferase suffices for the methylation reaction, whereas interaction along the full length of transcripts promotes transcription.

EXPERIMENTAL PROCEDURES

Antibodies

Antibodies were raised against full-length RNMT and RAM (Gonatopoulos-Pournatzis et al., 2011). Others were raised against actin (ab3280, Abcam); LEO1 (A300-174A-T, Bethyl Laboratories); phosphor-S2 Pol II (3E10) and phospho-S5 Pol II (3E8, Chromotek); CTR9 (12619S), H2Bub (5546P), H3K4me3 (9727S), and H3K36me3 (9763S, Cell Signaling Technology); Ac-H3 (06-599, Millipore); anti-HA horseradish peroxidase (HRP) conjugate (12013819001, Roche); and RNA Pol II (sc-899) and H3 (sc-10809, Santa Cruz Biotechnology).

Cell Culture

HeLa and HEK293 cells were cultured in DMEM and 10% fetal bovine serum at 37°C in 5% CO_2. 46C mESCs were cultured on 0.1% gelatin-coated plates in Glasgow's minimum essential medium (GMEM), 10% knockout serum replacement, 1% mem-NEAA, 1 mM sodium pyruvate, 0.1 mM 2-mercaptoethanol, and 100 U/mL recombinant Leukemia inhibitory factor (LIF). Transient transfections were performed using Lipofectamine 2000 (Thermo Fisher Scientific) with 4 μg pcDNA5, 2 μg pcDNA5-HA-RNMT, 4 μg pcDNA5-HA-RNMT Δ416–456, 2 μg pcDNA5 HA-RNMT MTD, 2 μg pcDNA5-HA-RNMT Δ1–120, and 1 μg pcDNA5-HA-RNMT 1–120 or 4 μg of pcDNA5 RAM-GFP for 48 hr. siRNA transfections were performed with 200 pmol RAM siRNA or non-targeting control (siGenome, Dharmacon) using RNAiMAX (Thermo Fisher Scientific) for 36 or 48 and 72 hr in HeLa and mESCs, respectively. siRAM1 and siRAM4 in HeLa cells are D-021286-01 and D-021286-04 (Dharmacon). The siRAM mixture used in mESCs is equimolar D-049592-01 and D-049592-03 (Dharmacon). HeLa and mESCs stably expressing RNMT were made with the pBMN retroviral vector system and pPyPCAGGS vector. Cells were treated with 3 μg/mL of actinomycin D or DMSO for RNA decay experiments.

CLIP

CLIP was performed according to Huppertz et al. (2014) with the following alterations. Five to ten 100 mm plates with subconfluent HeLa cells were UV-crosslinked with 250 mJ/cm^2 at 254 nm in Stratalinker 2400. Lysates were sonicated for five 30 s pulses in a Bioruptor (Diagenode) at low amplitude. Nuclease digestion with 5 μL Turbo DNase (Life Technologies) and 10 μL 1:500 (high) to 1:10,000 (low) dilution of RNase A (70194Z, Affymetrix) were performed in a thermomixer at 37°C for 3 min of shaking at 1,100 rpm. HA-RNMT was immunoprecipitated with 10 μL Pierce anti-HA magnetic beads for 2 hr at 4°C. IP RNA was labeled using the ^{32}P-labeled 3′-RNA linker (5′-Pmn.GGAACCGUGGGCUCUUAAGGU-3′) prepared by kinase labeling and purification through Sephadex G-25 columns as in Moore et al. (2014). Reverse transcription and linearization were performed using RT primer and Cut oligo as mentioned in Table S1. Sequencing for CLIP was performed on the Illumina MiSeq platform. Reads were aligned with Novoalign (Novocraft Technologies), with duplicates collapsed using tools from the cross-induced mutation sites (CIMS) pipeline and reads per transcript counted with HTSeq (Anders et al., 2015; Moore et al., 2014). Genome coverage was calculated using BedTools, and the read distribution profile was plotted with ngs.plot, with combined data for all replicates (Quinlan, 2014; Shen et al., 2014). Input sequencing, alignment, and quantification are described in the RNA-Seq section. RPKM and CPM values were obtained from the edgeR package (Robinson et al., 2010). Correlations with UTR length and input counts were performed with custom R scripts. RNMT-enriched transcripts were

determined by intersecting the top quartiles of three replicates with a cutoff of 50 counts per million aligned reads. Gene Ontology analysis was performed on the Bioconductor GOseq package (Young et al., 2010).

ChIP

Chromatin immunoprecipitation (ChIP) was performed using 2 μg anti-RNA Pol II or 5 μL CTR9 antibody with 25 μg HeLa chromatin as in Varshney et al. (2015). Libraries for sequencing were prepared as in Wiechens et al. (2016), and sequencing was performed on Illumina NextSeq. ChIP reads were aligned with STAR, correlations were performed with deepTools, and the read distribution profile was plotted with ngs.plot (Dobin et al., 2013; Ramírez et al., 2014; Shen et al., 2014). Data from replicates were pooled for analysis of gene loci. Bedgraphs were visualized using the Integrated Genome Browser (Nicol et al., 2009). Fragments aligning uniquely to each annotated gene locus ± 2 kb, to each annotated TSS ± 1 kb, or to a gene body (TSS + 1 kb to TES + 2 kb) were counted with the HTSeq package (Anders et al., 2015). Only loci with more than 5 uniquely aligned counts per million per gene locus or 1 uniquely aligned count per million per TSS were considered for log fold change and p value calculations by exactTest in the Bioconductor edgeR package (Robinson et al., 2010). Peaks were called using MACS2 call summits, with a q value cutoff of 0.01.

IPs

Cell lysates were prepared in 50 mM Tris (pH 7.5), 50 mM sodium chloride, 1% Triton X-100, 270 mM sucrose, 1 mM sodium orthovanadate, 1 mM EGTA, 1 mM EDTA, 10 mM β-glycerol phosphate, 5 mM sodium pyrophosphate, 1 mM DTT, 10 μM leupeptin, 1 μM pepstatin, 0.1 mg/mL aprotinin, and phosphatase inhibitor (PI) 2 and PI3 cocktail (Sigma) and were precleared with 20 μL washed protein G Sepharose (GE Healthcare) for 30 min at 4°C on a rotating mixer. Resin was pelleted and supernatant was incubated with 2 μg antibody for 2 hr at 4°C. 1 μL RNase A (70194Z, Affymetrix) was added before IP where required. Protein G Sepharose beads were prepared by washing twice in lysis buffer, blocking with 1 mg/mL BSA at RT for 30 min, and washing thrice in lysis buffer. 20 μL of beads were added to each IP and incubated at 4°C for 1 hr on a rotating mixer. IPs were washed thrice with ice-cold lysis buffer without detergent and eluted with 2× Laemmli sample buffer by boiling at 70°C for 10 min before western blot analysis.

Nuclear Run-On

Nuclear run-on was performed as in Roberts et al. (2015) with the following modifications. 2 × 10^6 cells plated the day before were lysed and washed in buffer containing 10 μM leupeptin, 1 μM pepstatin, 0.1 mg/mL aprotinin, 1:1,000 phosphatase inhibitor cocktail II and III, and 5 mM DTT (Sigma). Nuclei were stored in nucleus storage buffer that contained the same protease and phosphatase inhibitors, DTT, and 1 U/μL RNAase inhibitor (RNAsin). 5fM recombinant RNMT and/or RAM protein were added to nuclei from a 10 cm plate and incubated for 20–30 min at 30°C, with 1 mM ATP, 1 mM cytidine trisphosphate (CTP), 1 mM guanosine triphosphate (GTP), 0.5 mM BrUTP, 0.5 mM UTP, and 1 U/μL RNAsin. Recombinant RAM 1–90 was used, rather than full-length RAM (1–118), because the latter is unstable. Following nuclear run-on and RNA purification, Br-U IP was performed with more than 10 μg RNA (normalized across samples). Following IP, RNA was resuspended in 30 μL, with 10 μL used for cDNA synthesis and PCR.

Quantification of Poly(A) RNA Synthesis by ^{3}H-Uridine Incorporation

Subconfluent HeLa cells were incubated with 60 μM 5,6-^{3}H-uridine (PerkinElmer) for 30 min 36–48 hr post-transfection with siRNA or pcDNA5-based constructs. Total RNA was purified with TRIzol (Thermo Fisher Scientific) and quantified using a Qubit RNA BR assay kit (Thermo Fisher Scientific). Poly(A) mRNA was purified from total RNA using the NEBNext poly(A) mRNA magnetic isolation module (NEB) or mRNA Direct (Ambion). ^{3}H-uridine incorporation into mRNA was quantified by scintillation, counting equal amounts (40–70 ng) of purified poly(A) RNA.

RNA-Seq

RNA was extracted from HeLa cells using the TRIzol reagent (Thermo Fisher Scientific). Libraries were prepared with TruSeq Stranded Total RNA with the Ribo-Zero kit (Illumina). Sequencing was performed on the Illumina NextSeq platform. Reads were aligned using STAR software, and transcripts were quantified with the HTSeq package (Anders et al., 2015; Dobin et al., 2013). Library normalization and differential expression analysis were performed using the Bioconductor edgeR package (Robinson et al., 2010). Only transcripts expressed above the threshold of 2 reads per million in 4+ replicates were considered for analysis. The p values were calculated using the GLMqlf test and adjusted by the Benjamini-Hochberg method. Data were plotted using custom R scripts.

RT-PCR

RNA was isolated using TRIzol (Thermo Fisher Scientific). 100–200 ng RNA was used to prepare cDNA using the iScript cDNA synthesis kit (Bio-Rad). PCR was performed with the EvaGreen supermix (Bio-Rad) on the CFX384 Touch real-time PCR detection system (Bio-Rad). Primer sequences are in Table S1.

SILAC Mass Spectrometry

HEK293 cells were cultured in R0K0 media with 84 mg/L L-arginine (Sigma) and 146 mg/L L-lysine (Sigma) or R4K4 media with an of equal concentration L-arginine 13C and l-Lysine 4,4,5,5-D4 (Cambridge Isotope Laboratories) for 5 cell doublings and were lysed in 10 mM HEPES, 15 mM $MgCl_2$, and 10 mM KCl. Lysates were precleared with protein G Sepharose. Equal cell lysates from R0K0- and R6K4-labeled 293 HA-RNMT cells were affinity purified with murine IgG agarose (Sigma) and monoclonal anti-HA antibody-conjugated agarose (Sigma), respectively, and washed with lysis buffer; eluates were mixed at a 1:1 ratio. The mix was resolved by SDS-PAGE, and lanes were excised into five slices before in-gel tryptic digestion. Samples were analyzed on a LTQ-Orbitrap XL mass spectrometer (Thermo Fisher Scientific) coupled to a U3000 nano-LC system (Dionex). Data were analyzed with MaxQuant (v.1.0.13.13) with the MaxQuant Human database and Mascot search engine v.2.3.2 (Matrix Science) (Cox and Mann, 2008; Cox et al., 2009). Data were filtered using the Protein Frequency library (Boulon et al., 2010).

DATA AND SOFTWARE AVAILABILITY

The accession number for the sequencing reported in this paper is GEO: GSE87767.

SUPPLEMENTAL INFORMATION

Supplemental Information includes nine figures, one table, and two data files and can be found with this article online at https://doi.org/10.1016/j.celrep.2018.04.004.

ACKNOWLEDGMENTS

We thank members of the Cowling lab, Tayside Centre for Genomic Analysis, and Data Analysis Group for discussions. We thank Alain-Pierre Petit for recombinant RNMT-RAM. Research was funded by a Medical Research Council Senior Non-Clinical Fellowship (MR/K024213/1), a Lister Prize Research Fellowship (to V.H.C.), a Wellcome Trust Centre Award (097945/Z/11/Z), and a Wellcome Trust Strategic Award (100476/Z/12/Z).

AUTHOR CONTRIBUTIONS

D.V., O.L., G.S., S.D., O.S., and V.H.C. performed the experiments and analyzed the data. D.V. and V.H.C. designed the project and wrote the manuscript.

DECLARATION OF INTERESTSS

The authors declare no competing interests.

Received: March 28, 2017
Revised: January 11, 2018
Accepted: March 30, 2018
Published: May 1, 2018

REFERENCES

Anders, S., Pyl, P.T., and Huber, W. (2015). HTSeq—a Python framework to work with high-throughput sequencing data. Bioinformatics *31*, 166–169.

Aregger, M., and Cowling, V.H. (2013). Human cap methyltransferase (RNMT) N-terminal non-catalytic domain mediates recruitment to transcription initiation sites. Biochem. J. *455*, 67–73.

Aregger, M., Kaskar, A., Varshney, D., Fernandez-Sanchez, M.E., Inesta-Vaquera, F.A., Weidlich, S., and Cowling, V.H. (2016). CDK1-Cyclin B1 Activates RNMT, Coordinating mRNA Cap Methylation with G1 Phase Transcription. Mol. Cell *61*, 734–746.

Battaglia, S., Lidschreiber, M., Baejen, C., Torkler, P., Vos, S.M., and Cramer, P. (2017). RNA-dependent chromatin association of transcription elongation factors and Pol II CTD kinases. eLife *6*, e25637.

Boulon, S., Ahmad, Y., Trinkle-Mulcahy, L., Verheggen, C., Cobley, A., Gregor, P., Bertrand, E., Whitehorn, M., and Lamond, A.I. (2010). Establishment of a protein frequency library and its application in the reliable identification of specific protein interaction partners. Mol. Cell. Proteomics *9*, 861–879.

Brannan, K., Kim, H., Erickson, B., Glover-Cutter, K., Kim, S., Fong, N., Kiemele, L., Hansen, K., Davis, R., Lykke-Andersen, J., and Bentley, D.L. (2012). mRNA decapping factors and the exonuclease Xrn2 function in widespread premature termination of RNA polymerase II transcription. Mol. Cell *46*, 311–324.

Buratowski, S. (2009). Progression through the RNA polymerase II CTD cycle. Mol. Cell *36*, 541–546.

Cox, J., and Mann, M. (2008). MaxQuant enables high peptide identification rates, individualized p.p.b.-range mass accuracies and proteome-wide protein quantification. Nat. Biotechnol. *26*, 1367–1372.

Cox, J., Matic, I., Hilger, M., Nagaraj, N., Selbach, M., Olsen, J.V., and Mann, M. (2009). A practical guide to the MaxQuant computational platform for SILAC-based quantitative proteomics. Nat. Protoc. *4*, 698–705.

Dermody, J.L., and Buratowski, S. (2010). Leo1 subunit of the yeast paf1 complex binds RNA and contributes to complex recruitment. J. Biol. Chem. *285*, 33671–33679.

Dobin, A., Davis, C.A., Schlesinger, F., Drenkow, J., Zaleski, C., Jha, S., Batut, P., Chaisson, M., and Gingeras, T.R. (2013). STAR: ultrafast universal RNA-seq aligner. Bioinformatics *29*, 15–21.

Ehrensberger, A.H., Kelly, G.P., and Svejstrup, J.Q. (2013). Mechanistic interpretation of promoter-proximal peaks and RNAPII density maps. Cell *154*, 713–715.

Furuichi, Y. (2015). Discovery of m(7)G-cap in eukaryotic mRNAs. Proc. Jpn. Acad., Ser. B, Phys. Biol. Sci. *91*, 394–409.

Furuichi, Y., LaFiandra, A., and Shatkin, A.J. (1977). 5′-Terminal structure and mRNA stability. Nature *266*, 235–239.

Glover-Cutter, K., Kim, S., Espinosa, J., and Bentley, D.L. (2008). RNA polymerase II pauses and associates with pre-mRNA processing factors at both ends of genes. Nat. Struct. Mol. Biol. *15*, 71–78.

Gonatopoulos-Pournatzis, T., and Cowling, V.H. (2014a). Cap-binding complex (CBC). Biochem. J. *457*, 231–242.

Gonatopoulos-Pournatzis, T., and Cowling, V.H. (2014b). RAM function is dependent on Kapβ2-mediated nuclear entry. Biochem. J. *457*, 473–484.

Gonatopoulos-Pournatzis, T., Dunn, S., Bounds, R., and Cowling, V.H. (2011). RAM/Fam103a1 is required for mRNA cap methylation. Mol. Cell *44*, 585–596.

Grasso, L., Suska, O., Davidson, L., Gonatopoulos-Pournatzis, T., Williamson, R., Wasmus, L., Wiedlich, S., Peggie, M., Stavridis, M.P., and Cowling, V.H. (2016). mRNA Cap Methylation in Pluripotency and Differentiation. Cell Rep. *16*, 1352–1365.

Groudine, M., Peretz, M., and Weintraub, H. (1981). Transcriptional regulation of hemoglobin switching in chicken embryos. Mol. Cell. Biol. *1*, 281–288.

Guiguen, A., Soutourina, J., Dewez, M., Tafforeau, L., Dieu, M., Raes, M., Vandenhaute, J., Werner, M., and Hermand, D. (2007). Recruitment of P-TEFb (Cdk9-Pch1) to chromatin by the cap-methyl transferase Pcm1 in fission yeast. EMBO J. *26*, 1552–1559.

Huppertz, I., Attig, J., D'Ambrogio, A., Easton, L.E., Sibley, C.R., Sugimoto, Y., Tajnik, M., König, J., and Ule, J. (2014). iCLIP: protein-RNA interactions at nucleotide resolution. Methods *65*, 274–287.

Jaehning, J.A. (2010). The Paf1 complex: platform or player in RNA polymerase II transcription? Biochim. Biophys. Acta *1799*, 379–388.

Kaneko, S., Chu, C., Shatkin, A.J., and Manley, J.L. (2007). Human capping enzyme promotes formation of transcriptional R loops *in vitro*. Proc. Natl. Acad. Sci. USA *104*, 17620–17625.

Kim, H.J., Jeong, S.H., Heo, J.H., Jeong, S.J., Kim, S.T., Youn, H.D., Han, J.W., Lee, H.W., and Cho, E.J. (2004). mRNA capping enzyme activity is coupled to an early transcription elongation. Mol. Cell. Biol. *24*, 6184–6193.

Kim, J., Guermah, M., and Roeder, R.G. (2010). The human PAF1 complex acts in chromatin transcription elongation both independently and cooperatively with SII/TFIIS. Cell *140*, 491–503.

Krebs, A.R., Imanci, D., Hoerner, L., Gaidatzis, D., Burger, L., and Schubeler, D. (2017). Genome-wide Single-Molecule Footprinting Reveals High RNA Polymerase II Turnover at Paused Promoters. Mol. Cell *67*, 411–422.

Mandal, S.S., Chu, C., Wada, T., Handa, H., Shatkin, A.J., and Reinberg, D. (2004). Functional interactions of RNA-capping enzyme with factors that positively and negatively regulate promoter escape by RNA polymerase II. Proc. Natl. Acad. Sci. USA *101*, 7572–7577.

Moore, M.J., Zhang, C., Gantman, E.C., Mele, A., Darnell, J.C., and Darnell, R.B. (2014). Mapping Argonaute and conventional RNA-binding protein interactions with RNA at single-nucleotide resolution using HITS-CLIP and CIMS analysis. Nat. Protoc. *9*, 263–293.

Nicol, J.W., Helt, G.A., Blanchard, S.G., Jr., Raja, A., and Loraine, A.E. (2009). The Integrated Genome Browser: free software for distribution and exploration of genome-scale datasets. Bioinformatics *25*, 2730–2731.

Nilson, K.A., Lawson, C.K., Mullen, N.J., Ball, C.B., Spector, B.M., Meier, J.L., and Price, D.H. (2017). Oxidative stress rapidly stabilizes promoter-proximal paused Pol II across the human genome. Nucleic Acids Res. *45*, 11088–11105.

Orphanides, G., and Reinberg, D. (2002). A unified theory of gene expression. Cell *108*, 439–451.

Pei, Y., Du, H., Singer, J., Stamour, C., Granitto, S., Shuman, S., and Fisher, R.P. (2006). Cyclin-dependent kinase 9 (Cdk9) of fission yeast is activated by the CDK-activating kinase Csk1, overlaps functionally with the TFIIH-associated kinase Mcs6, and associates with the mRNA cap methyltransferase Pcm1 *in vivo*. Mol. Cell. Biol. *26*, 777–788.

Perales, R., and Bentley, D. (2009). "Cotranscriptionality": the transcription elongation complex as a nexus for nuclear transactions. Mol. Cell *36*, 178–191.

Pillutla, R.C., Yue, Z., Maldonado, E., and Shatkin, A.J. (1998). Recombinant human mRNA cap methyltransferase binds capping enzyme/RNA polymerase IIo complexes. J. Biol. Chem. *273*, 21443–21446.

Quinlan, A.R. (2014). BEDTools: The Swiss-Army Tool for Genome Feature Analysis. Curr. Protoc. Bioinformatics *47*, 11.12.1–11.12.34.

Ramanathan, A., Robb, G.B., and Chan, S.H. (2016). mRNA capping: biological functions and applications. Nucleic Acids Res. *44*, 7511–7526.

Ramírez, F., Dündar, F., Diehl, S., Grüning, B.A., and Manke, T. (2014). deepTools: a flexible platform for exploring deep-sequencing data. Nucleic Acids Res. *42*, W187–W191.

Roberts, T.C., Hart, J.R., Kaikkonen, M.U., Weinberg, M.S., Vogt, P.K., and Morris, K.V. (2015). Quantification of nascent transcription by bromouridine immunocapture nuclear run-on RT-qPCR. Nat. Protoc. *10*, 1198–1211.

Robinson, M.D., McCarthy, D.J., and Smyth, G.K. (2010). edgeR: a Bioconductor package for differential expression analysis of digital gene expression data. Bioinformatics *26*, 139–140.

Saldi, T., Cortazar, M.A., Sheridan, R.M., and Bentley, D.L. (2016). Coupling of RNA Polymerase II Transcription Elongation with Pre-mRNA Splicing. J. Mol. Biol. *428*, 2623–2635.

Schroeder, S.C., Schwer, B., Shuman, S., and Bentley, D. (2000). Dynamic association of capping enzymes with transcribing RNA polymerase II. Genes Dev. *14*, 2435–2440.

Schroeder, S.C., Zorio, D.A., Schwer, B., Shuman, S., and Bentley, D. (2004). A function of yeast mRNA cap methyltransferase, Abd1, in transcription by RNA polymerase II. Mol. Cell *13*, 377–387.

Schwartz, B.E., Larochelle, S., Suter, B., and Lis, J.T. (2003). Cdk7 is required for full activation of *Drosophila* heat shock genes and RNA polymerase II phosphorylation *in vivo*. Mol. Cell. Biol. *23*, 6876–6886.

Shen, L., Shao, N., Liu, X., and Nestler, E. (2014). ngs.plot: Quick mining and visualization of next-generation sequencing data by integrating genomic databases. BMC Genomics *15*, 284.

Shilatifard, A. (2008). Molecular implementation and physiological roles for histone H3 lysine 4 (H3K4) methylation. Curr. Opin. Cell Biol. *20*, 341–348.

Shuman, S. (2015). RNA capping: progress and prospects. RNA *21*, 735–737.

Topisirovic, I., Svitkin, Y.V., Sonenberg, N., and Shatkin, A.J. (2011). Cap and cap-binding proteins in the control of gene expression. Wiley Interdiscip. Rev. RNA *2*, 277–298.

Varshney, D., Vavrova-Anderson, J., Oler, A.J., Cowling, V.H., Cairns, B.R., and White, R.J. (2015). SINE transcription by RNA polymerase III is suppressed by histone methylation but not by DNA methylation. Nat. Commun. *6*, 6569.

Varshney, D., Petit, A.P., Bueren-Calabuig, J.A., Jansen, C., Fletcher, D.A., Peggie, M., Weidlich, S., Scullion, P., Pisliakov, A.V., and Cowling, V.H. (2016). Molecular basis of RNA guanine-7 methyltransferase (RNMT) activation by RAM. Nucleic Acids Res. *44*, 10423–10436.

Wade, P.A., Werel, W., Fentzke, R.C., Thompson, N.E., Leykam, J.F., Burgess, R.R., Jaehning, J.A., and Burton, Z.F. (1996). A novel collection of accessory factors associated with yeast RNA polymerase II. Protein Expr. Purif. *8*, 85–90.

Wagschal, A., Rousset, E., Basavarajaiah, P., Contreras, X., Harwig, A., Laurent-Chabalier, S., Nakamura, M., Chen, X., Zhang, K., Meziane, O., et al. (2012). Microprocessor, Setx, Xrn2, and Rrp6 co-operate to induce premature termination of transcription by RNAPII. Cell *150*, 1147–1157.

Wiechens, N., Singh, V., Gkikopoulos, T., Schofield, P., Rocha, S., and Owen-Hughes, T. (2016). The Chromatin Remodelling Enzymes SNF2H and SNF2L Position Nucleosomes adjacent to CTCF and Other Transcription Factors. PLoS Genet. *12*, e1005940.

Yang, E., van Nimwegen, E., Zavolan, M., Rajewsky, N., Schroeder, M., Magnasco, M., and Darnell, J.E., Jr. (2003). Decay rates of human mRNAs: correlation with functional characteristics and sequence attributes. Genome Res. *13*, 1863–1872.

Yao, X., Tang, Z., Fu, X., Yin, J., Liang, Y., Li, C., Li, H., Tian, Q., Roeder, R.G., and Wang, G. (2015). The Mediator subunit MED23 couples H2B mono-ubiquitination to transcriptional control and cell fate determination. EMBO J. *34*, 2885–2902.

Young, M.D., Wakefield, M.J., Smyth, G.K., and Oshlack, A. (2010). Gene ontology analysis for RNA-seq: accounting for selection bias. Genome Biol. *11*, R14.

Yu, M., Yang, W., Ni, T., Tang, Z., Nakadai, T., Zhu, J., and Roeder, R.G. (2015). RNA polymerase II-associated factor 1 regulates the release and phosphorylation of paused RNA polymerase II. Science *350*, 1383–1386.

Cell Reports, Volume *23*

Supplemental Information

mRNA Cap Methyltransferase, RNMT-RAM, Promotes RNA Pol II-Dependent Transcription

Dhaval Varshney, Olivia Lombardi, Gabriele Schweikert, Sianadh Dunn, Olga Suska, and Victoria H. Cowling

Figure S1

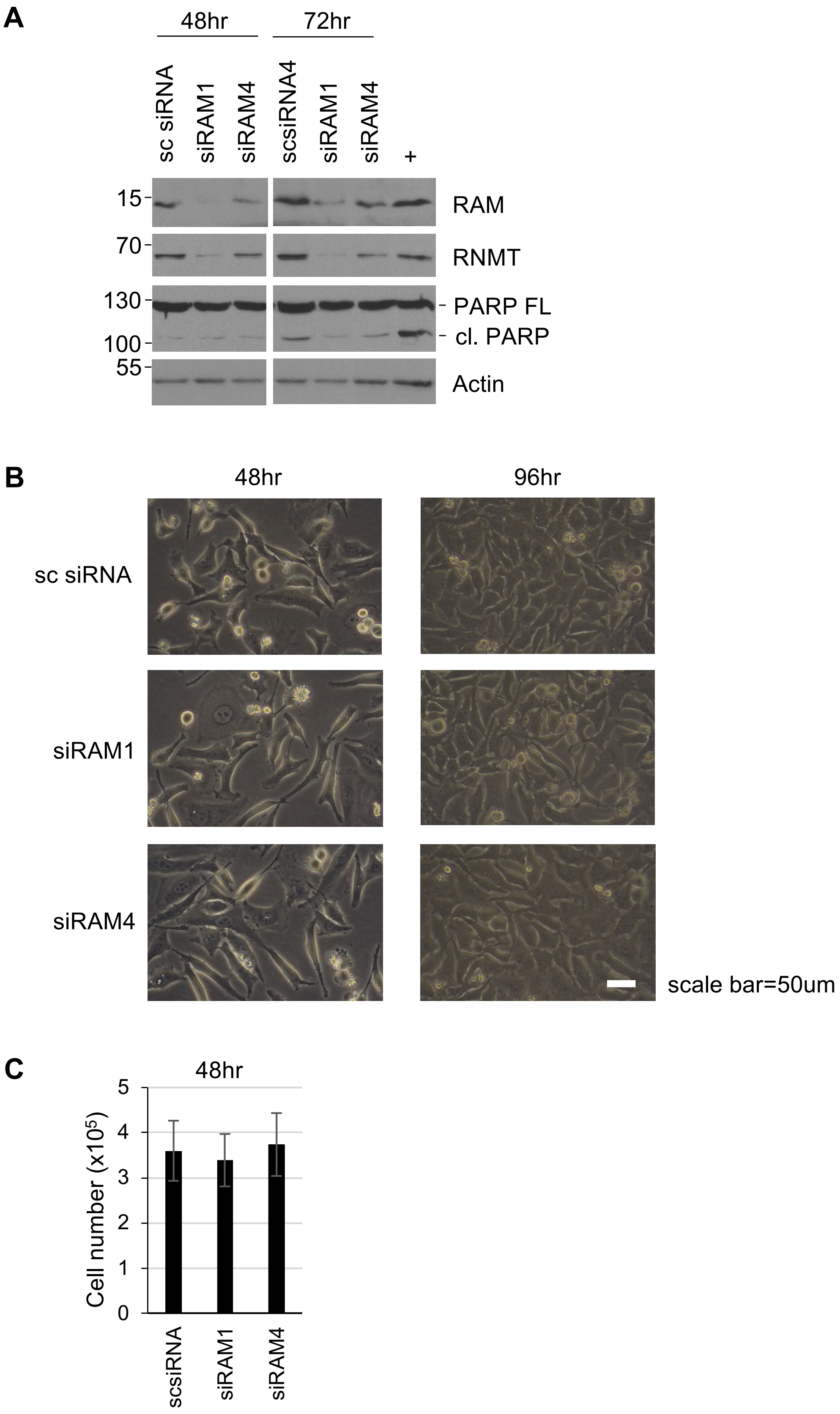

Figure S1, related to Figure 1: Impact of RAM depletion on cell number and apoptosis
HeLa cells were transfected with siRAM1, siRAM4 and sc siRNA (scrambled control). After time indicated, a) RAM expression and cleavage of PARP assessed by western blot. "+" indicates cells treated with 250um THZ-1 for 2 hours to induce apoptosis (representative of 2 independent experiments), b) phase-contrast images taken at 400X magnification (representative of 2 independent experiments), c) cells were counted (average and standard error presented for 3 independent experiments).

Figure S2

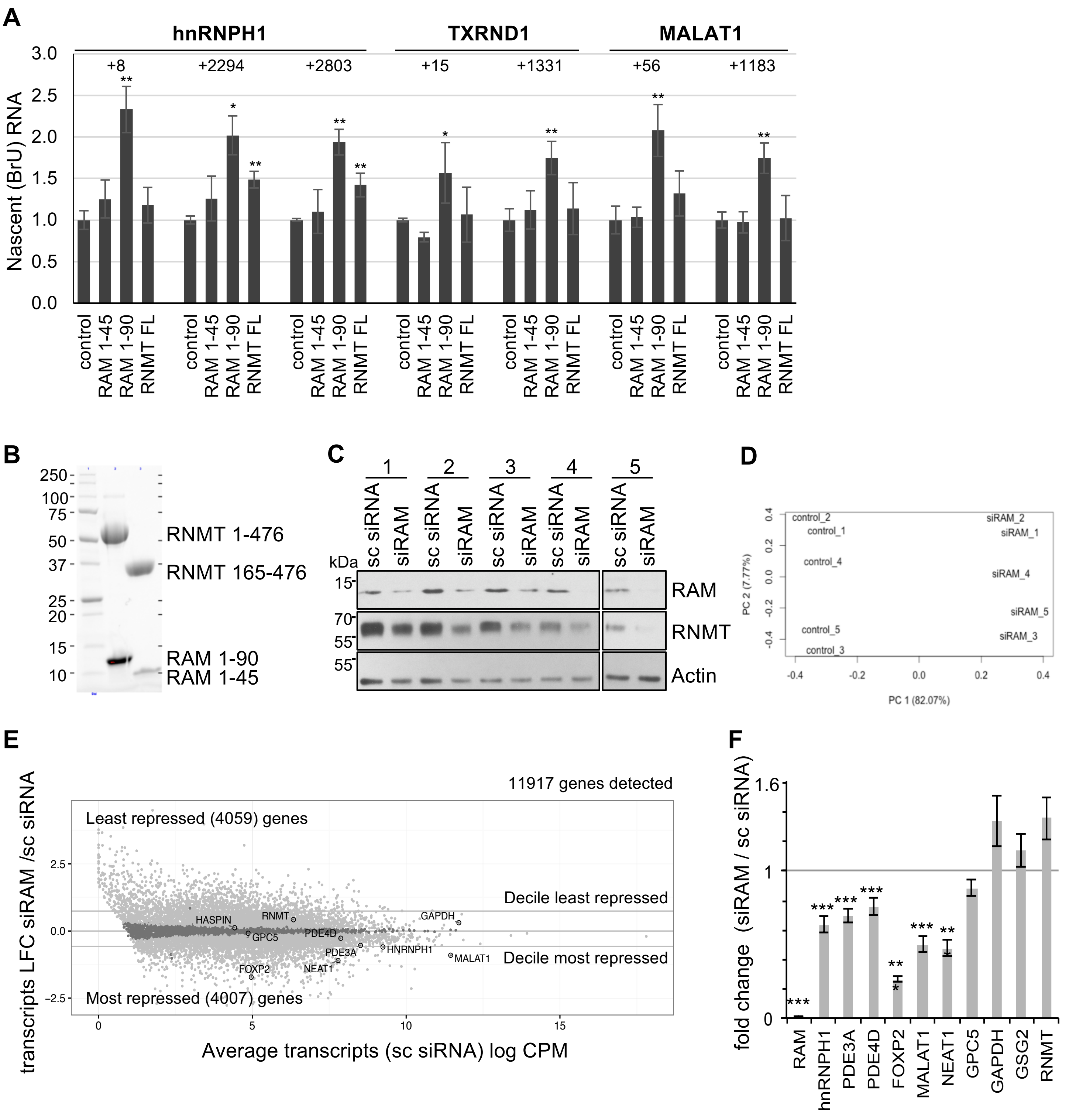

Figure S2, related to Figure 1. Impact of RAM on transcription.

a) HeLa nuclei were incubated with NTPs, BrUTP and recombinant RNMT, RAM 1-90 or RAM 1-45 for 20mins. Br-RNA purified and used as a substrate for PCR with primers indicated. Average and standard deviation presented. n=4. *, P < 0.05; **; P <0.01; Students t-test. b) SDS-PAGE analysis of recombinant RNMT-RAM proteins used in this study. c) HeLa cells transfected with siRAM 1 or sc siRNA for 36 hours, in five independent biological replicates. Western blot analysis. d) Principal components analysis of RNA sequencing performed. e) RNA sequencing analysis. Scatter plot of differential transcript expression following RAM siRNA or sc siRNA transfection for 36hrs (n=5). For each annotated gene locus, $\log_2$ fold change (LFC siRAM/sc siRNA) expression plotted against expression in control cells ($\log_2$ counts per million (logCPM) of uniquely aligned reads). Deciles most and least repressed indicated. Most repressed genes () and least repressed genes shaded lighter and annotated. f) HeLa cells transfected with siRAM1 or sc siRNA for 36hrs. RTPCR analysis of mRNA indicated following . n=3 ***, P <0.005; Students t-test.

Figure S3

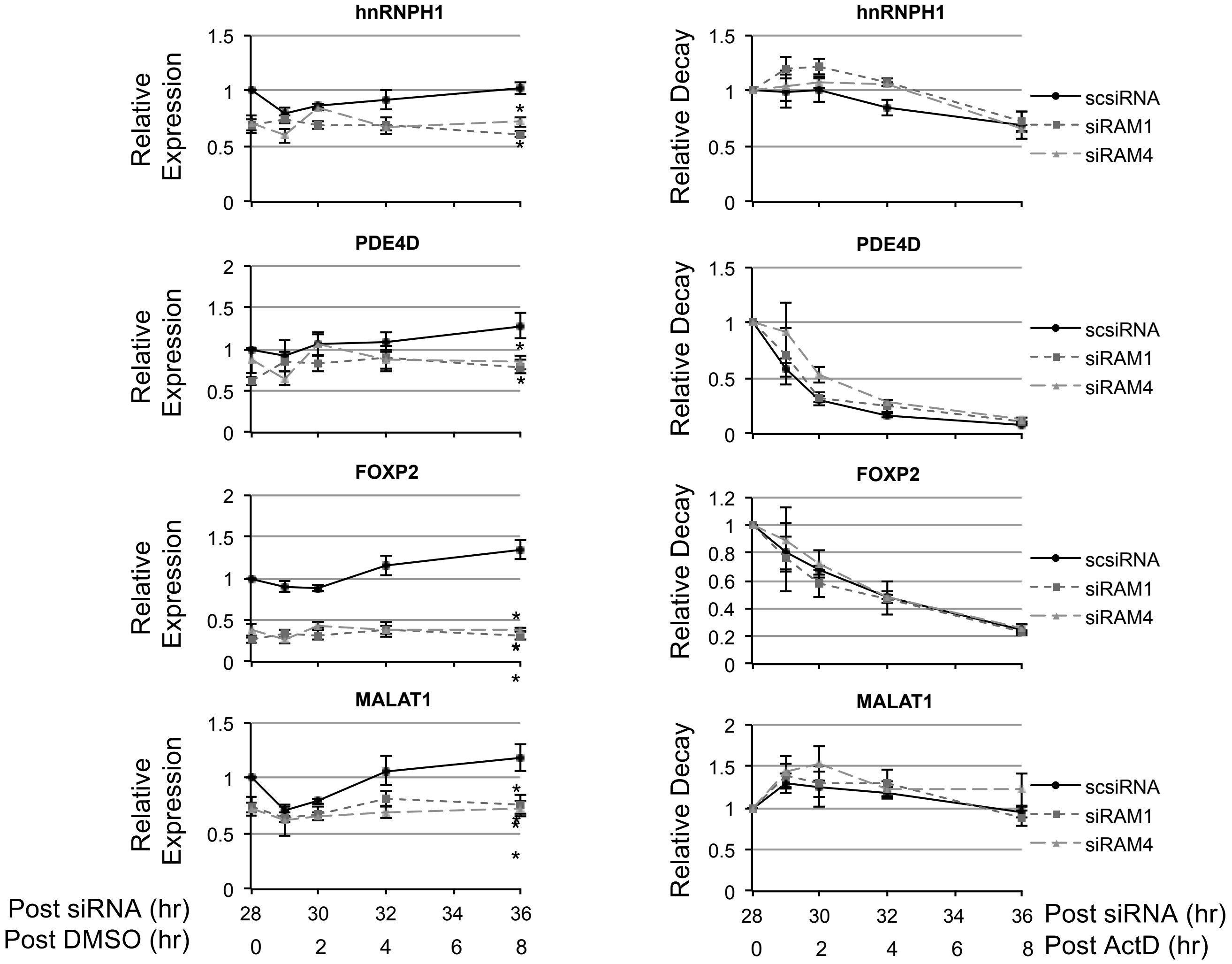

Figure S3, related to Figure 1. Gene expression analysis following RAM siRNA transfection
Hela cells transfected with RAM siRNA or non-targeting control. 28 hours post-transfection 3μg/μl Actinomycin D or DMSO control added for further 8 hours. Mature transcript level determined by RTPCR. DMSO control data (left panels) normalised to 0 hour scrambled control. Actinomycin D data normalised to 0 hour samples for each siRNA (right panels).

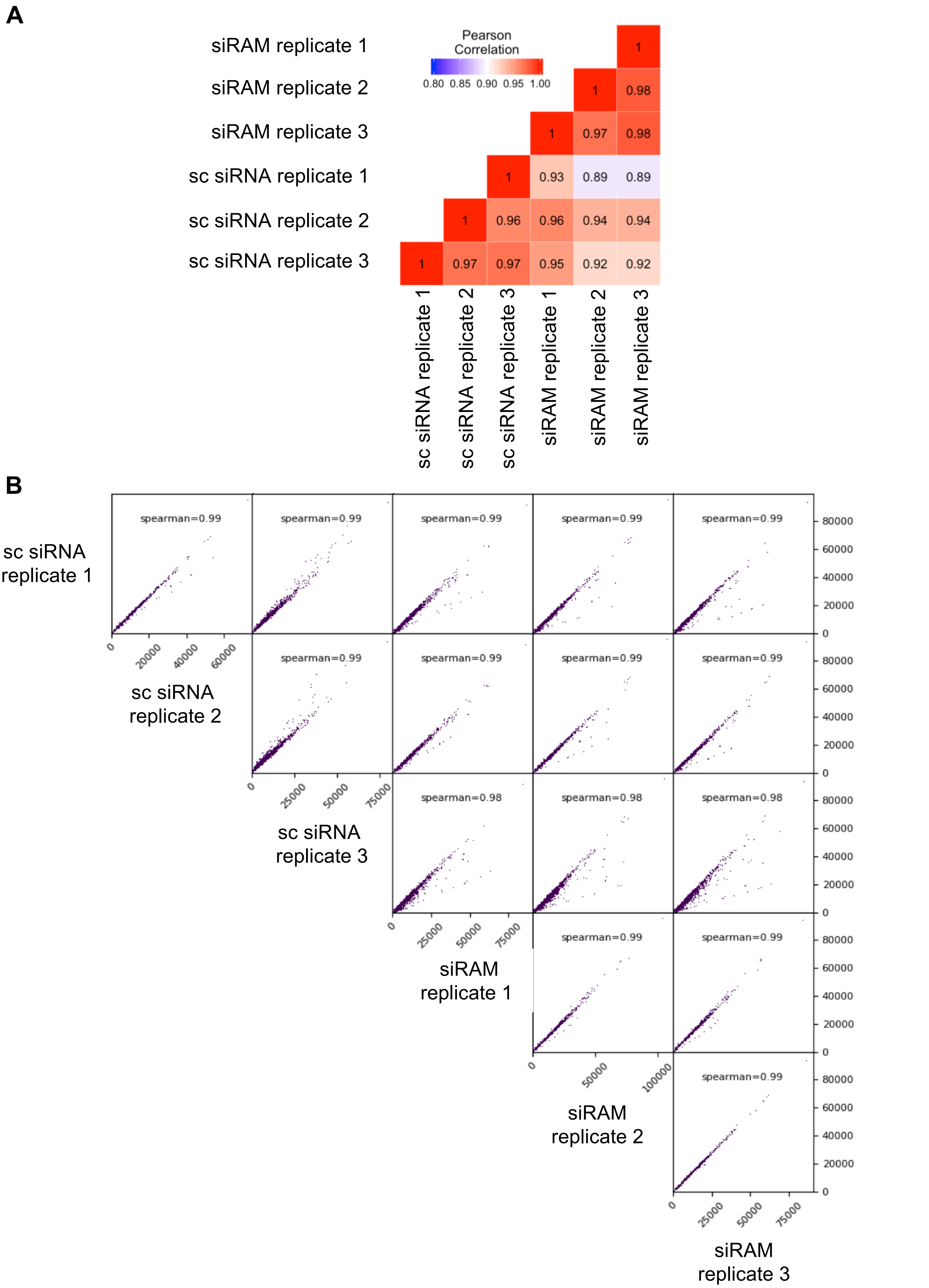

Figure S4, related to Figure 2. Correlation of RNA Pol II ChIP-Seq replicates

A) Heatmap for Pearson correlation of individual RNA pol II ChIP replicates from HeLa cells transfected with siRAM 1 or sc siRNA for 36 hours. Genome wide correlation for tag alignment to 10Kb bins. B) Scatterplots for RNA pol II ChIP replicates showing the total number of tags uniquely aligning to individual annotated loci. Calculated Spearman correlation values shown.

Figure S5

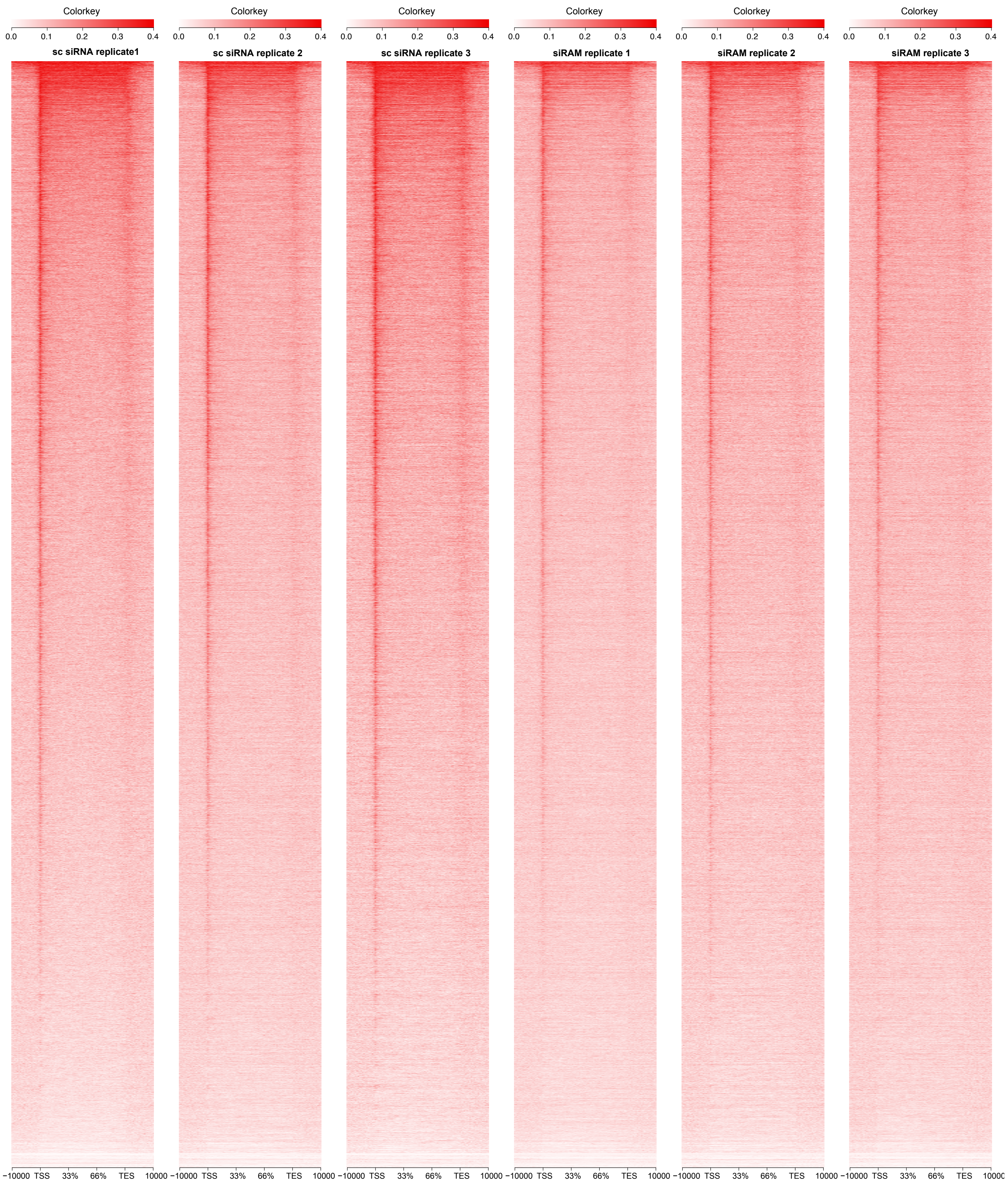

Figure S5, related to Figure 2. Heatmaps for RNA pol II ChIP replicates over gene bodies
Gene body heatmaps for all annotated gene loci in human genome assembly hg38 produced using ngs.plot (Shen et al., 2014)

Figure S6

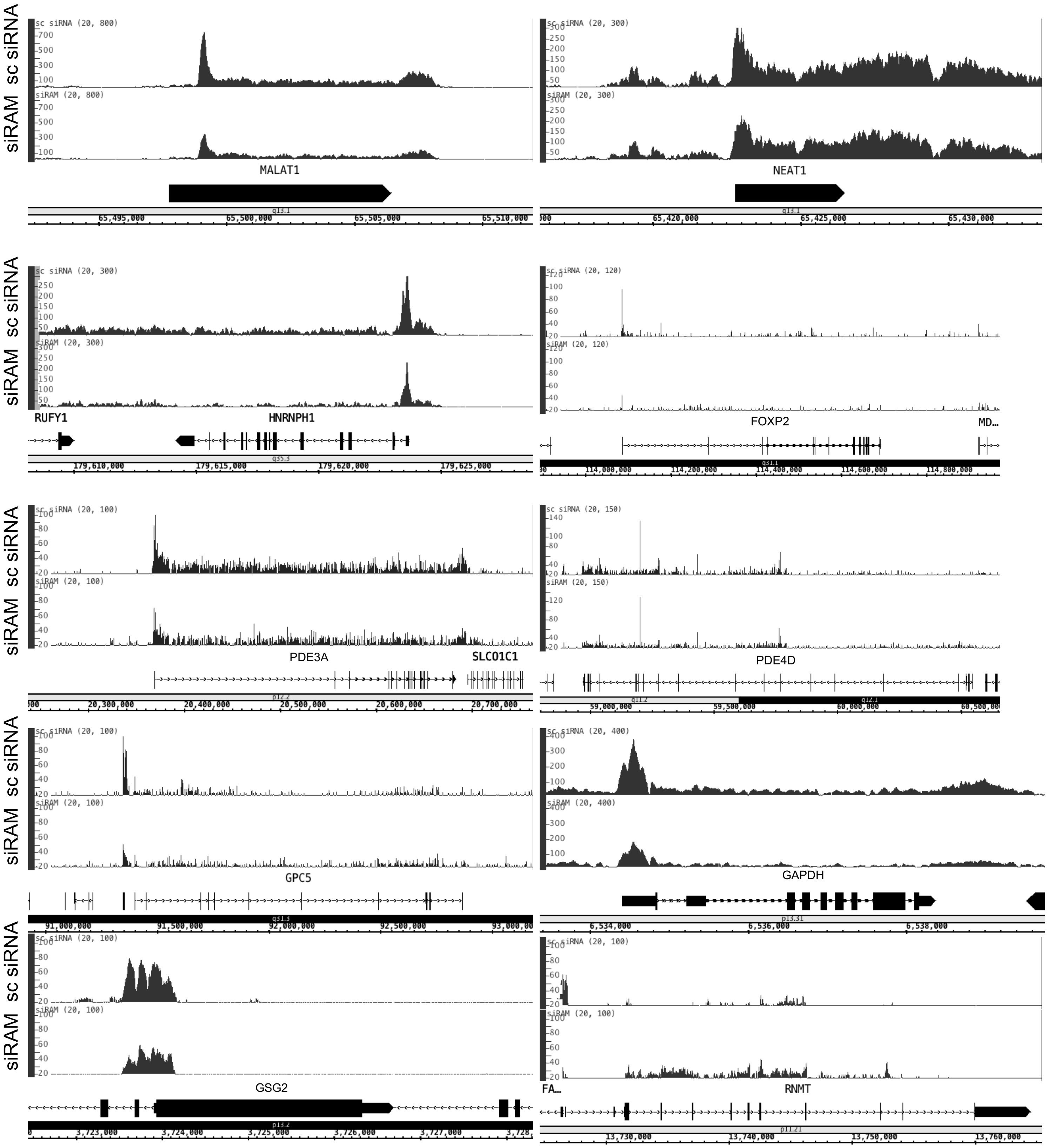

Figure S6, related to Figure 2. RNA Pol II ChIP signal at individual gene loci
Snapshot of RNA pol II ChIP tag distribution over individual gene loci following sc siRNA or si RAM transfection. Profiles created using pooled data from three replicates. Minimum cutoff of 20 tags.

Figure S7

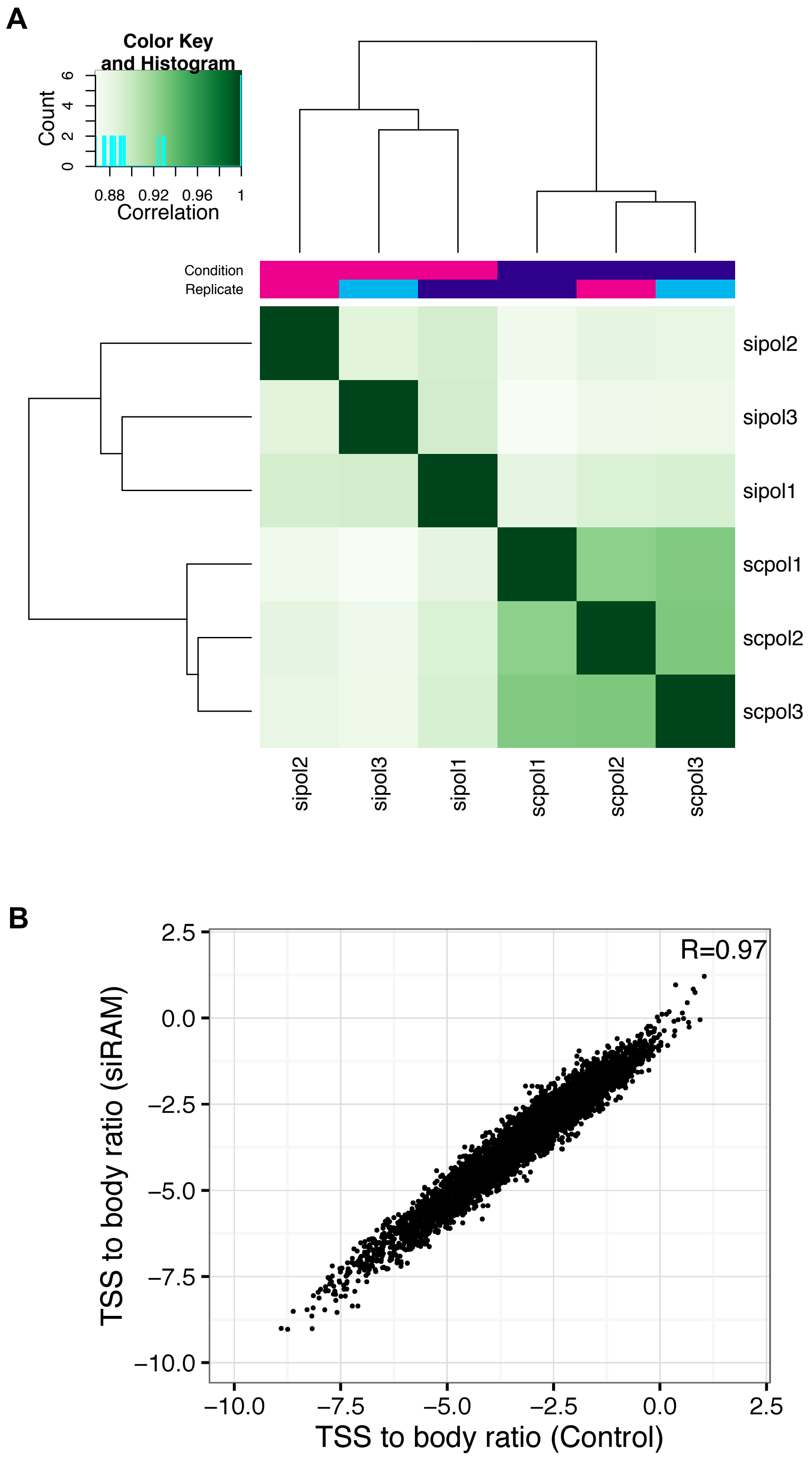

Figure S7, related to Figure 2. RAM depletion results in RNA pol II depletion
a) Correlation heatmap for peaks called from three independent biological replicates for RNA pol II occupancy in sc siRNA (sc) and siRAM (si) transfected cells. b) Correlation of Log_2 CPM TSS/gene body ratios of RNA pol II occupancy in sc siRNA (control) and siRAM transfected cells. R value represents Pearson's correlation.

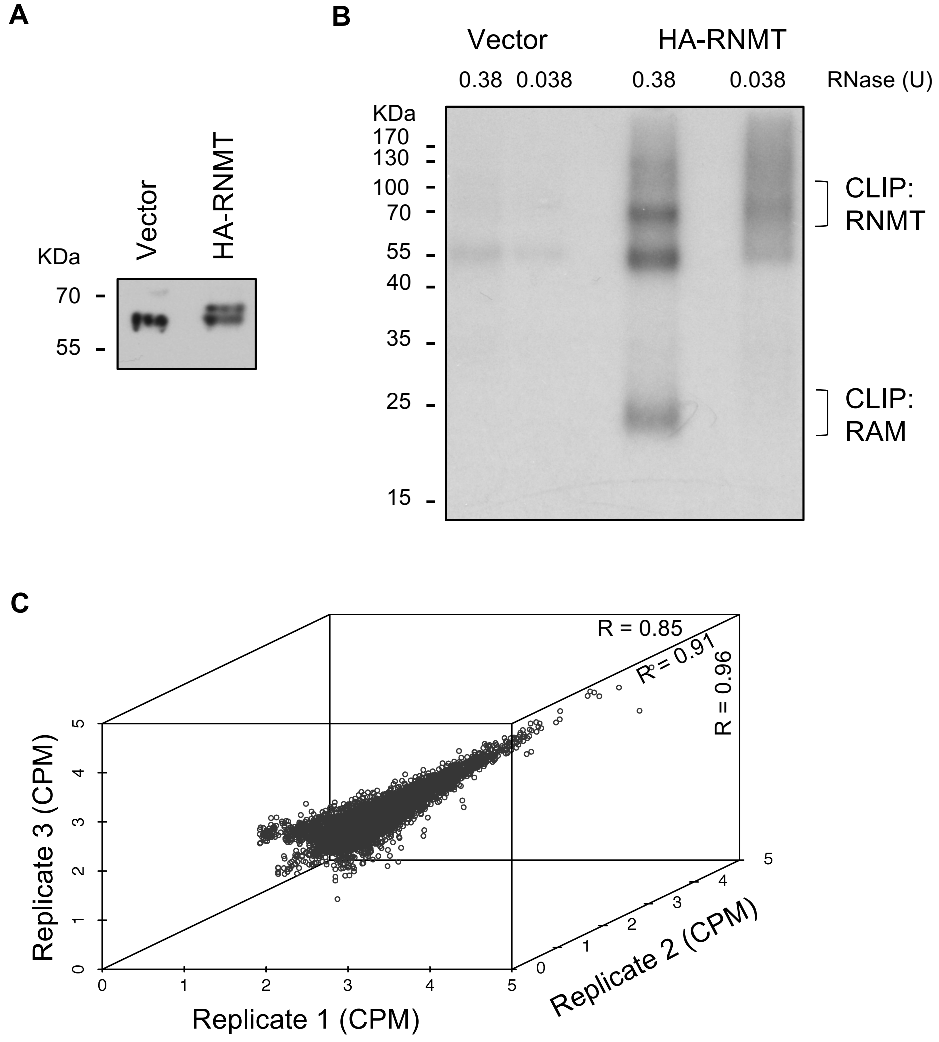

Figure S8, related to Figure 3. HA-RNMT expression and RNMT-CLIP

a) Western blot analysis of RNMT expression in HA-RNMT-expressing and vector control HeLa cells. The lower band is endogenous RNMT, the upper band is HA-RNMT. b) CLIP assay performed on HeLa cells stably expressing HA-RNMT or vector control. Immunoprecipitation performed with anti-HA antibody after RNase treatment. Reduced RNase concentration resulted in smearing of the ^{32}P-RNMT band and reduced intensity of the ^{32}P-RAM band. RAM is likely to be tethered to RNMT via RNA, migrating in a large RNMT-RAM-RNA complex. RNA from smear between 70 and 100 kDa was isolated for sequencing. c) Following RNA sequencing analysis, scatter plot of total counts per million (CPM) uniquely aligned reads per annotated gene locus, for three HA-RNMT CLIP replicates. Correlation scores on xy, yz and xz planes.

Figure S9

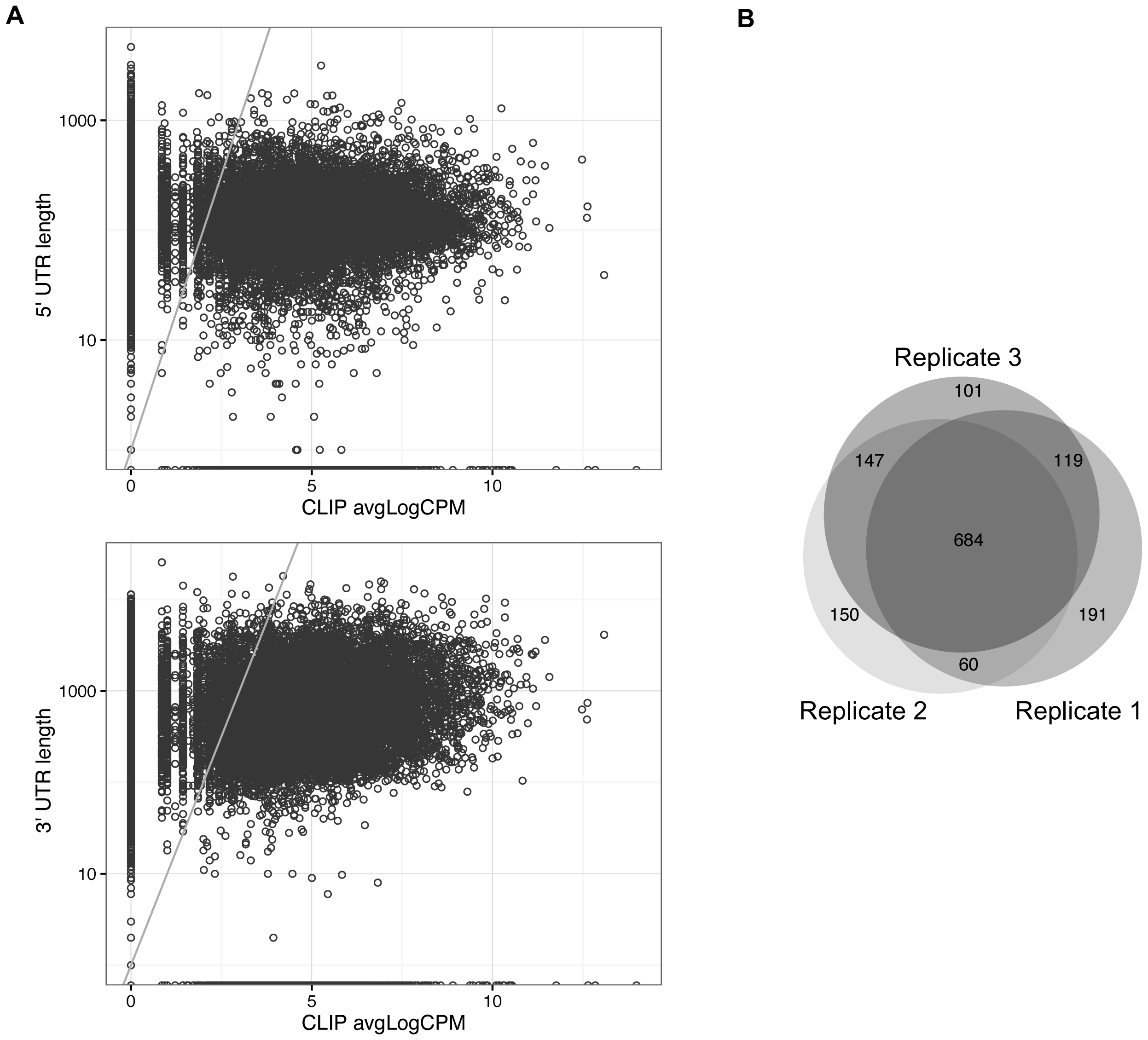

Figure S9, related to Figure 6. Correlation of RNMT CLIP signal and UTR length
a) Average Log_2 CPM from RNMT CLIP signal from three independent biological replicates plotted against the maximal annotated UTR length for each gene model. b) Venn diagram of top quartiles for three RNMT CLIP replicates. Intersection of annotated gene loci with maximum number of uniquely aligned reads denoted "RNMT-enriched targets".

Table S1, related to Figures 1 and 5. Oligo sequences used for RT-qPCR analysis and CLIP library preparation

Target	Primer	Sequence
Actin	F	CCCAGAGCAAGAGAGG
Actin	R	GTCCAGACGCAGGATG
FOXP2	F	CACTTCCTGTCCAATCGCTG
FOXP2 mature	R	TTGGAGGAGGTAGTCGAGGA
FOXP2 nascent	R	GCCACCATGTTAACAATCACG
GAPDH	F	GGAGTCAACGGATTTGG
GAPDH	R	GTAGTTGAGGTCAATGAAGGG
GPC5	F	GGAGAGATATCAGATTGCGGC
GPC5	R	ACATAACACGGCCAGTCCTT
Haspin	F	GTCCGACGATCCTGACGAT
Haspin	R	TTTGGGGTCACGGTCAGG
hnRNPH1	F	GGTGTTGAAGCATACTGGTCC
hnRNPH1 mature	R	GTCCACCGGCAATGTTATCC
hnRNPH1 nascent	R	GCTAGCCAAAACTCACTGCT
MALAT1	F	GACGGAGGTTGAGATGAAGC
MALAT1	R	ATTCGGGGCTCTGTAGTCCT
NEAT1	F	TTGTTCCAGAGCCCATGAA
NEAT1	R	TGAAAACCTTTACCCCAGGA
PDE3A	F	CAGCAGCAAGTCCCATCG
PDE3A mature	R	CTGTGATCCTCTTGGCCCTC
PDE3A nascent	R	CTTCCCTTTAGTTCCCAGCG
PDE4D	F	CATGTGCAACCAACCATCCA
PDE4D mature	R	TAGGGTCTCTAGCTGGTCCA
PDE4D nascent	R	TGACTCTTGGCAGAAATGGT
RAM	F	CCTCAAACCTTTGGGATT
RAM	R	TTCCTGATACTCCTTGTCA
RNMT	F	GTACTGATATTGCCGATGT
RNMT	R	ATTCACTATCACGACGATT
CLIP - RT Primer		CNNNNGATCGTCGGACTGTAGAACTCTG AACGGATCCCTTGGCACC
Cut Oligo		CCAAGGGATCCGTTCAGAGTTCTAAAA
HNRNPH1 NRO 8-77	F	ACCTTATTTAGGTTGCGCAG
HNRNPH1 NRO 8-77	R	ACCTAGACACGCGACTTCTG
HNRNPH1 NRO 2294-2363	F	AACGGTTGTTTTCTTTCCAGACT
HNRNPH1 NRO 2294-2363	R	TCTGGTGTAGATGAAACGAATACCT
HNRNPH1 NRO 2803-2873	F	GTTCAGTTCTTCTCAGGTATGTAGT
HNRNPH1 NRO 2803-2873	R	CCTTGCCATAAGCTAGCCAAA
MALAT1 56-126	F	TTTTAGCAACGCAGAAGCCC
MALAT1 56-126	R	GAGGCGTCAGAGGGGAC
MALAT1 1183-1253	F	ACTACTTTTTGCCTCCCTCAC
MALAT1 1183-1253	R	ACGGAGAACAACTCGCATCA
TXNRD1 15-85	F	CTTGTGCGACATGGGCTG
TXNRD1 15-85	R	TTTCGTCTGCAGCTCCGTT
TXNRD1 1331-1401	F	CAAGGTCAGGCAAGGTCCA

TXNRD1 1331-1401	R	CCTGCTCAAACTCCTTCATTGT
HNRNPH1+1	F	GTGGGCTGGCTCTTGAAATT
HNRNPH1+1	R	GGCTAAGACGAAATGGCCAG
HNRNPH1+500	F	CTCTCAGTTCTGGTCCTCCC
HNRNPH1+500	R	GTGCCCAACATCATCGTCTC
HNRNPH1+1000	F	GCAGGTTTGGCAGACTTTGT
HNRNPH1+1000	R	AGCTTGGGTGACAGAGACTC
FOXP2+1	F	CTTTGTCACCCCTCACGTTG
FOXP2+1	R	GTGATTGTTTTGGCGTCCGT
FOXP2+500	F	AGAGAGGGGTGGGATTTTACG
FOXP2+500	R	GCTTTTCCCTCGCAAACAGA
FOXP2+1000	F	AGAACCTGGGATCCTATTGGG
FOXP2+1000	R	CCCTCATAGCTTGTTCATGCA
PDE3A+1	F	TGAGCGTGCTAGCCTTTAAC
PDE3A+1	R	GCCCACAATCCACTGAAAGT
PDE3A+500	F	TTCTGGATGGGCTTGTACCT
PDE3A+500	R	CGCTAGTCAAGGCGATCAT
PDE3A+1000	F	GAGGGAACAGGTAAGCACTG
PDE3A+1000	R	CTTCCCTTTAGTTCCCAGCG
GAPDH+1	F	TACTAGCGGTTTTACGGGC
GAPDH+1	R	GCTGCGGGCTCAATTTATAG
GAPDH+500	F	GAACCGCGTCTACGAGC
GAPDH+500	R	CTGACCTTGAGCTCTCCTTG
GAPDH+1000	F	GATTTCTCCTCCGGGTGATG
GAPDH+1000	R	CCAACTACCCATGACTCAGC
